# Regulating Integrin β1 to Restore Gonadotropin-Releasing Hormone–Tanycyte Unit Function in Polycystic Ovary Syndrome-Related Hypothalamic Dysregulation

**DOI:** 10.34133/research.0619

**Published:** 2025-02-19

**Authors:** Yu Wang, Xiaoyu Tong, Yan Xiao, Yicong Wang, Wei Hu, Wenhan Lu, Yuning Chen, Jiajia Li, Wenhao Gao, Hongru Gao, Yicheng Tian, Sizhe Dai, Yi Feng

**Affiliations:** ^1^ Department of Integrative Medicine and Neurobiology, School of Basic Medical Sciences, State Key Laboratory of Medical Neurobiology and MOE Frontiers Center for Brain Science, Institutes of Brain Science, Fudan University, Shanghai 200032, China.; ^2^ Shanghai Key Laboratory of Acupuncture Mechanism and Acupoint Function, Shanghai Institute of Acupuncture and Moxibustion, Shanghai 200433, China.; ^3^ Department of Ophthalmology & Visual Science, Eye & ENT Hospital, Shanghai Medical College, Fudan University, Shanghai, China.

## Abstract

Excessive gonadotropin-releasing hormone (GnRH) is considered to be an initiating factor in the etiology of polycystic ovary syndrome (PCOS). GnRH neuronal axons terminate at the hypothalamic arcuate nucleus and median eminence, where tanycytes, specialized glial cells, have been proposed to modulate GnRH secretion through plasticity. However, the precise role of the “GnRH-tanycyte unit” during the pathological state of PCOS has not been thoroughly explored. In this study, we demonstrated the architecture and distribution of GnRH neurons and tanycytes. In PCOS-like mice, retracted tanycyte processes and dysregulated GnRH-tanycyte unit may create an environment conducive to the excessive secretion of GnRH and subsequent reproductive endocrine dysfunction. Mechanistically, excessive androgens impair hypothalamic neuroglial homeostasis by acting through the androgen receptor (AR) and its downstream target integrin β1 (Itgb1), thereby suppressing the FAK/TGF-βR1/Smad2 signaling pathway. Both selective deletion of *AR* and overexpression of *Itgb1* in tanycytes counteracted the detrimental effects of androgens, alleviating endocrine dysfunction. Collectively, this study highlights the alterations in the GnRH-tanycyte unit mediated by androgen/AR/Itgb1 signaling and provides a novel perspective for developing therapies for hypothalamic hormone secretion disorders by maintaining solid neuroglial structures in the brain.

## Introduction

Polycystic ovary syndrome (PCOS) is a prevalent endocrine and reproductive disorder characterized by irregular ovulation, hyperandrogenism, and the presence of polycystic ovary morphology [1,2]. Emerging evidence highlights the hypothalamus as a critical regulator of neuroendocrine dysfunction in PCOS, with disruptions in hypothalamic gonadotropin-releasing hormone (GnRH) secretion playing a pivotal role in the etiology of the syndrome [3]. GnRH is essential for regulating sexual maturation and endocrine functions by stimulating the anterior pituitary to release luteinizing hormone (LH) [4,5]. Although previous studies have identified GnRH hyperactivity as a prominent characteristic of PCOS, the underlying mechanisms regulating GnRH production and secretion remain incompletely understood.

GnRH neurons establish extensive networks of projections from the hypothalamic preoptic area to the capillary network in the basal hypothalamus [6]. The GnRH neuron axon terminals are enclosed by specialized ependymoglial cells known as tanycytes [7]. Tanycytes serve as physical sheaths that envelop hormone-producing neuron terminals, thereby regulating the secretion of neurohormones into the pituitary portal system [8–10]. The morphological plasticity of tanycyte processes can modify their spatial relationship with adjacent GnRH neurons at the neurovascular junction, potentially influencing GnRH release and mediating feedback from peripheral hormones to hypothalamic GnRH neurons [11]. Dysregulation of the GnRH-tanycyte unit may therefore represent a critical factor in the pathophysiology of PCOS. We hypothesize that the GnRH-tanycyte unit undergoes both morphological and functional alterations in PCOS, thereby contributing to excessive GnRH secretion and subsequent disruptions in peripheral reproductive function.

Barbotin and colleagues [12,13] demonstrated that anti-Müllerian hormone promotes the morphological remodeling of tanycyte processes and GnRH neuron terminals, suggesting that irregularities in circulating gonadal steroids may contribute to the pathology of the GnRH-tanycyte unit and accompanying reproductive dysfunction. Elevated androgen levels are a hallmark clinical feature and a key diagnostic criterion for patients with PCOS [14], and increased central androgen signaling through the androgen receptor (AR) is closely involved in the aberrant expression and secretion of GnRH [15,16]. Therefore, androgen signaling could potentially influence the GnRH-tanycyte unit. As a key regulator of cell adhesion, migration, and cytoskeletal organization, integrin β1 (Itgb1) is essential for maintaining tissue morphogenesis and cellular homeostasis in neuroglial systems [17]. Its involvement in neuronal–glial interactions suggests a potential role in regulating hypothalamic tanycytes and GnRH neurons [18,19]. Additionally, focal adhesion kinase (FAK) participates in integrin-mediated signal transduction, activating transforming growth factor-β receptor 1 (TGF-βR1) and its downstream effector Smad2 [20,21]. These physiological characteristics and functions make Itgb1 a pivotal candidate for exploring molecular mechanisms underlying abnormal neuroglial homeostasis in PCOS. However, few studies have examined the association between the Itgb1 signaling pathway and the GnRH-tanycyte unit [9].

This study used 3-dimensional visualization methods to examine the intricate architecture of GnRH neurons and tanycyte processes within the hypothalamus. By employing transgenic mice for *AR* knockdown and *Itgb1* overexpression in tanycytes, a critical molecular mechanism governed by the androgen-related AR/Itgb1 pathway was identified. The results provide a panoramic view of the impaired GnRH-tanycyte unit in PCOS and highlight potential therapeutic targets for treating neuroendocrine disorders.

## Results

### Three-dimensional reconstruction of the GnRH-tanycyte unit in the hypothalamus

**Fig. 1.**
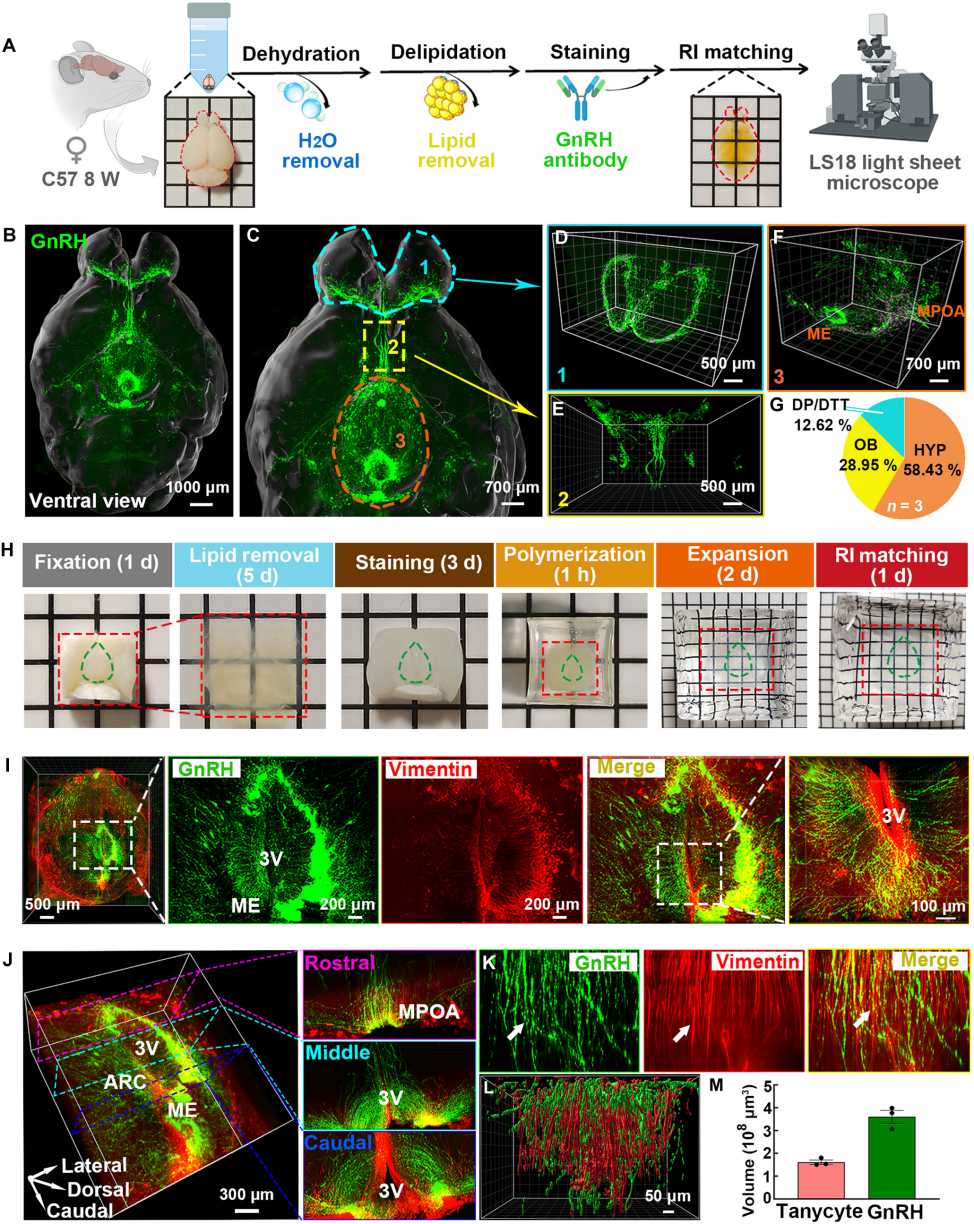
Reconstructed 3-dimensional images of GnRH neurons (green) and tanycytes (red). (A) Schematic representation of the iDISCO method. Following a series of processes, including dehydration, delipidation, and antibody staining, the whole mouse brain was subjected to 3-dimensional imaging using a light sheet microscope. (B and C) Images of whole mouse brain immunostaining with GnRH antibody. Three-dimensional view showing the distribution of GnRH neurons in the olfactory bulb (D), the dorsal peduncular cortex and the dorsal tenia tecta (E), and the hypothalamus (F). Cell bodies were identified using the Spots algorithm in the Imaris software (purple). (G) Pie chart showing the proportion of GnRH-positive signals across various brain regions (*n* = 3). (H) Schematic representation of the cMAP method for clearing and expanding the mouse hypothalamus. The duration of each step is indicated. The areas encircled by green lines denote the region near the 3V. (I) Three-dimensional images of GnRH axons and tanycytes were obtained from the hypothalamus. GnRH neuronal axons (visualized by GnRH antibody) formed branching structures close to tanycyte processes (visualized by vimentin antibody). (J and K) Three-dimensional rendering and slice view of GnRH neuronal axons and tanycyte process crosstalk in the rostral, middle, and caudal region of the hypothalamus. (L) Reconstructed images of GnRH (green) and tanycytes (red). (M) The relative fluorescence signal intensity of GnRH and tanycytes was reconstructed using Imaris software.

Whole brains from adult female mice were rendered transparent using the immunolabeling-enabled 3-dimensional imaging of solvent-cleared organs (iDISCO) method (Fig. 1A). Subsequent image reconstruction revealed that GnRH neurons were widely distributed throughout the brain (Fig. 1B and C and Fig. S1). Using the brain region alignment software BIRDSplug, we successfully aligned the whole-brain imaging data with the Allen Brain Atlas to ascertain the distribution of GnRH neurons in distinct brain regions (Fig. S2). Reconstructed images and statistical analysis of the fluorescence signals obtained from GnRH neurons revealed their predominant localization in the hypothalamus (HYP; 58.43%), followed by the olfactory bulb (OB; 28.95%), and the dorsal peduncular cortex and the dorsal tenia tecta (DP/DTT; 12.62%; Fig. 1D to G). Within the hypothalamus, GnRH neuron cell bodies were primarily localized in the medial preoptic area (MPOA), with their axons arranged in a looping pattern and projecting predominantly to the arcuate nucleus (ARC) and median eminence (ME). This spatial arrangement was further validated using GnRH-CreERT2;Ai14 transgenic mice, which facilitated the specific labeling of GnRH neurons, thereby corroborating the dual immunolabeling for tdTomato and GnRH antibodies (Fig. S3A and B). The 3-dimensional imaging of the hypothalamus in GnRH-Ai14 mice confirmed the presence of a dense network of GnRH axons within the ARC and ME (Fig. S3C).

Considering the previously identified potential role of tanycytes in regulating GnRH release, the hypothalami of mice were expanded and rendered transparent to better elucidate the spatial configuration and anatomical relationships within the GnRH-tanycyte unit. The clearing and magnification analysis of proteome (cMAP) technique was used to proportionally enlarge the hypothalamus 3-fold to improve the image resolution (Fig. 1H). Three-dimensional imaging revealed that tanycyte processes extended into the hypothalamic parenchyma, forming close connections with the axon terminals of GnRH neurons (Fig. 1I). We compiled images from multiple slices to observe the distribution of tanycyte processes and GnRH neuron axons around the third ventricle (3V), confirming the wrapping of GnRH neurons by tanycytes (Fig. 1J and K). The fluorescence signals of GnRH and tanycytes were quantified simultaneously (Fig. 1L and M).

### Mice with PCOS-like traits exhibit dysregulated GnRH-tanycyte unit

**Fig. 2.**
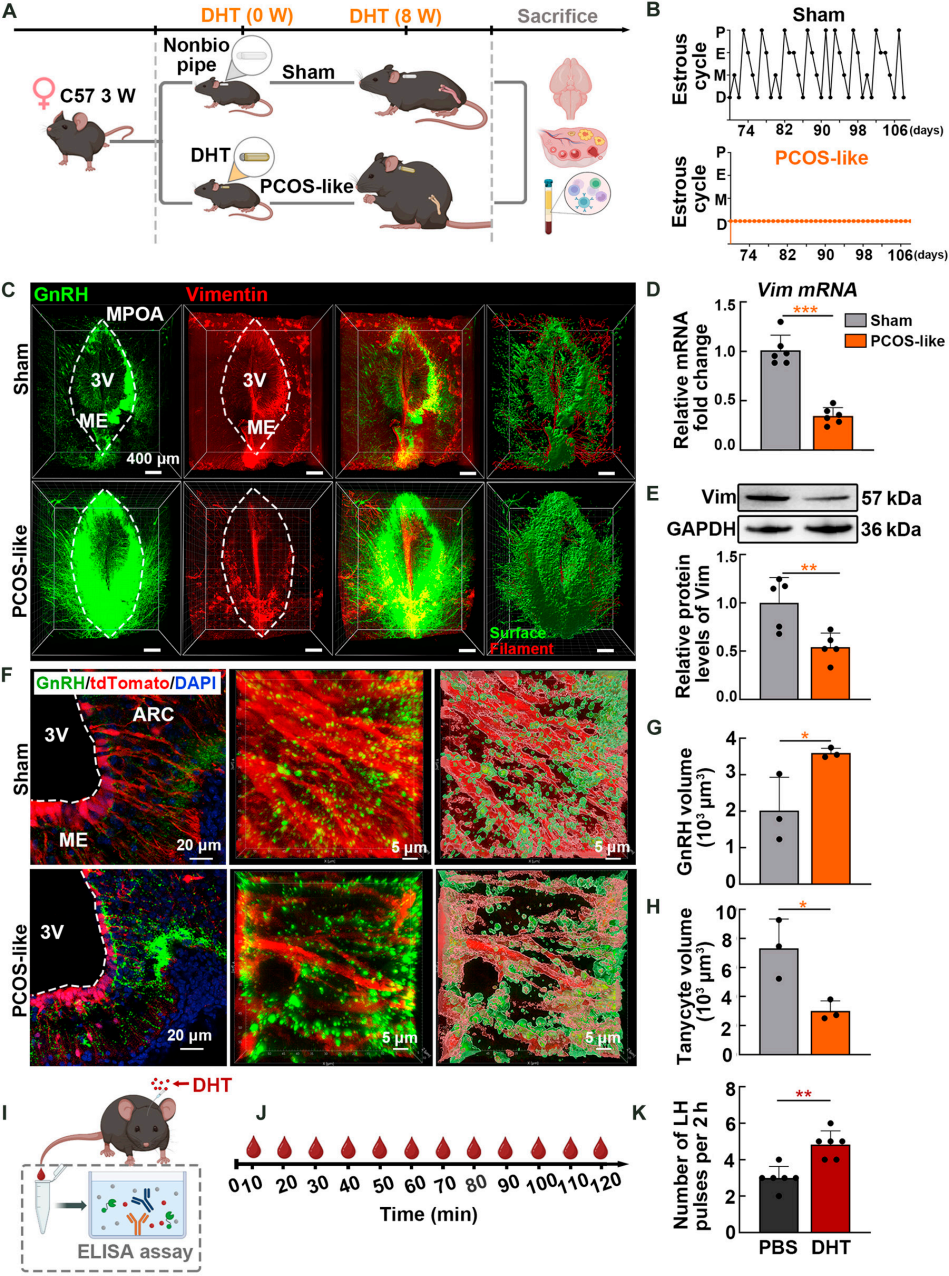
PCOS-like mice showed dysregulated GnRH-tanycyte unit in the hypothalamus. (A) Schematic of the experimental design of the PCOS-like mouse model. (B) Representative estrous cycles of mice in the sham and PCOS groups. Proestrus (P), estrus (E), metestrus (M), and diestrus (D) (*n* = 5, the estrous cycle detection lasted for 39 d). (C) Original and reconstructed positive signals of GnRH and tanycytes using the cMAP clearing method. Vimentin is frequently used as a marker to delineate tanycyte cytoskeleton morphology. The areas circled by white lines indicate the regions where tanycyte processes were enriched. Surfaces (green, labeling of GnRH neuron axons) and filaments (red, labeling of tanycyte processes) algorithms reconstructed in the Imaris software represent fluorescence signal volumes. (D) Real-time PCR analysis of *Darpp32* mRNA in the hypothalamus of sham and PCOS-like mice (*n* = 6, unpaired Student’s *t* test, *** *P* < 0.001). (E) Representative WB showing vimentin expression in the hypothalamus (*n* = 5, unpaired Student’s *t* test, **P < 0.01). (F) Representative fluorescence images of the ARC and ME in the hypothalamus. In the Rax-CreERT2;Ai14 mice, tanycyte processes were labeled in red, while GnRH was labeled in green. The areas were derived from the external zone of the ME, where neuroendocrine terminals come into proximity with the pericapillary space. Reconstruction of the positive signals was performed using the Surface automation algorithm of the Imaris software. (G and H) Quantification of GnRH and tanycyte process volumes reconstructed using the Surface automation algorithm of the Imaris software (*n* = 3, unpaired Student’s *t* test, *P < 0.05). (I and J) Schematic representation of tail-tip blood sample collection in adult diestrus female mice (3 to 4 months old) and the ELISA assay. (K) Representative number of LH pulses after receiving PBS and DHT injection (*n* = 6, unpaired Student’s *t* test, **P < 0.01).

The dihydrotestosterone (DHT; a typical androgen)-induced PCOS-like mouse model was established, and the reproductive endocrine functions of the mice were evaluated (Fig. 2A). Mice in the PCOS group exhibited disrupted estrous cycles, altered levels of sex hormones, and a reduced number of preovulatory follicles and corpora lutea, confirming the successful construction of the PCOS-like model (Fig. 2B and Fig. S4). It is noteworthy that the GnRH antagonist cetrorelix acetate mitigated the symptoms of PCOS, emphasizing the role of excessive GnRH in the development of DHT-induced PCOS (Fig. S5).

To further investigate the central mechanisms responsible for the excessive GnRH secretion, 3-dimensional images were obtained to evaluate changes in the GnRH-tanycyte unit within the expanded hypothalamic region surrounding the 3V. Androgen-induced PCOS-like mice showed abundant GnRH-positive fluorescence signals and reduced tanycyte markers (Fig. 2C) as well as a decrease in both *Darpp32* mRNA and vimentin (Vim) protein levels following PCOS modeling (Fig. 2D and E). The retina and anterior neural fold homeobox transcription factor (Rax) is selectively expressed in tanycytes located at the 3V [22]. Rax-CreERT2;Ai14 transgenic mice were used to observe tanycyte processes in detail because of the expression of tdTomato fluorescent protein under the control of tanycyte-specific Cre recombinase. Substantial changes in tanycyte processes were observed, with PCOS-like mice displaying diminished tanycyte processes near the basal lamina, followed by up-regulation of GnRH (Fig. 2F). Statistical analysis of GnRH and tanycyte process volumes further confirmed these observations. In PCOS-like mice, the GnRH-positive signal showed a marked increase, indicating enhanced GnRH axon aggregation, while the tanycyte process volume significantly decreased, reflecting impaired structural integrity of the tanycytes (Fig. 2G and H). Additionally, we assessed the area occupied by tanycyte processes following cetrorelix treatment and found that, although PCOS symptoms improved, the tanycyte processes did not revert to the phenotype observed in control animals (Fig. S6).

We further investigated how hormonal signals orchestrated the aberrant changes in tanycyte processes. Female mice were intraventricularly injected with dissolved DHT during the diestrus stage, and immunostaining was performed for the immediate early genes c-Fos and vimentin. In response to excessive androgen, tanycytes showed a robust response with increased c-Fos and reduced process volume, while these factors remained largely unchanged in phosphate-buffered saline (PBS)-injected mice (Fig. S7). Pulsatile LH serves as an alternative indicator for hypothalamic GnRH pulse frequency [23,24], and mice injected with DHT exhibited increased LH pulsatility over time compared with those in the control group (Fig. 2I to K). These results demonstrate that androgen exposure notably disrupts the balance and normal functioning of the GnRH-tanycyte unit, potentially leading to hormonal dysregulation.

### AR antagonists restore tanycyte process expression and reproductive endocrine capacity

**Fig. 3.**
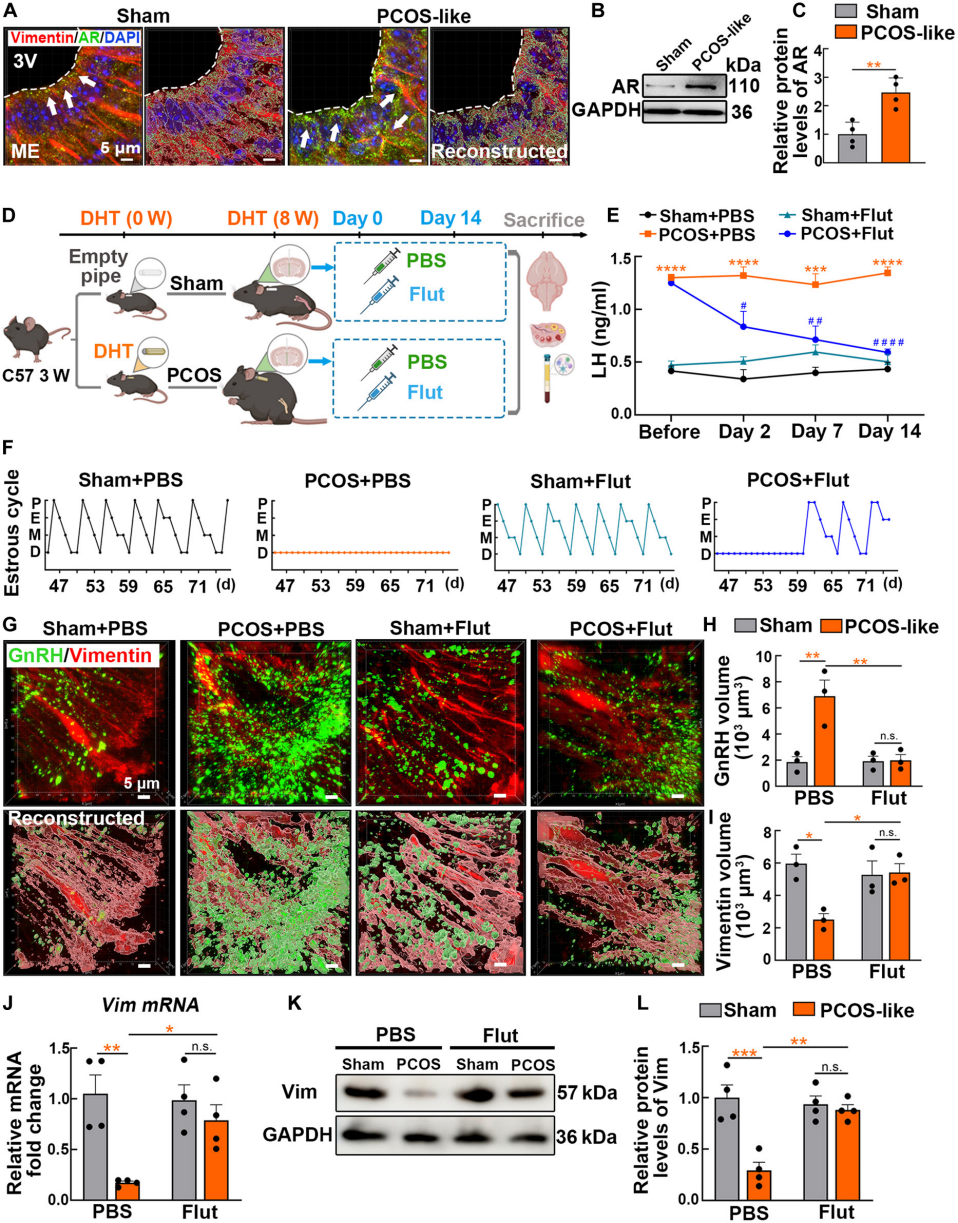
The AR antagonist Flut restored the neuroendocrine function and morphology of tanycyte processes in PCOS-like mice. (A) Representative high-resolution images showing vimentin (red) and AR (green) immunoreactivities near the 3V. The positive signals were reconstructed using the Surface algorithm of the Imaris software. (B and C) Relative protein expression of AR in the sham and PCOS groups (*n* = 4, unpaired Student’s *t* test, **P < 0.01). (D) Experimental design diagram of DHT and Flut administration. After establishing the PCOS model, a total of 1 μl of Flut (1 mM) was injected into the 3V for 14 d (once every other day). (E) ELISA measured LH levels in tail-blood samples before drug administration and on days 2, 7, and 14 after PBS or Flut injection at the same time (8:00 AM to 10:00 AM) (*n* = 4, 2-way ANOVA, Tukey’s post hoc test, ***P < 0.001, ****P < 0.0001 versus sham + PBS group; ^#^*P* < 0.05, ^##^*P* < 0.01, ^####^*P* < 0.0001 versus PCOS + PBS group). (F) Representative mouse estrous cycle in the sham + PBS, PCOS + PBS, sham + Flut, and PCOS + Flut groups over 32 d. (G) Representative immunofluorescence images of tanycyte processes (red) and GnRH (green) in the basal layer of the hypothalamus. Reconstruction of the positive signals was performed using the Surface automation algorithm of the Imaris software. (H and I) Quantification of GnRH and vimentin volume reconstructed using the Surface automation algorithm of the Imaris software (*n* = 3, 2-way ANOVA followed by Tukey’s post hoc test, *P < 0.05 and **P < 0.01). (J) Real-time PCR analysis of *Darpp32* mRNA expression in the sham + PBS, PCOS + PBS, sham + Flut, and PCOS + Flut groups (*n* = 4, 2-way ANOVA followed by Tukey’s post hoc test, *P < 0.05 and **P < 0.01). (K and L) Representative bands and grayscale values of vimentin protein expression in the hypothalamus for each group of mice (*n* = 4, 2-way ANOVA followed by Tukey’s post hoc test, **P < 0.01 and ***P < 0.001).

Because AR is the principal receptor for androgens, we conducted further investigations into the alterations in hypothalamic AR expression in PCOS-like mice. Comprehensive findings from high-resolution immunofluorescence imaging, polymerase chain reaction (PCR), and Western blot (WB) analyses consistently demonstrated a marked increase in AR-positive signals near the 3V (Fig. 3A to C and Fig. S8A). The classical AR antagonist flutamide (Flut) was administered to functionally characterize the role of hypothalamic AR in GnRH secretion. Hypothalamic explants containing the ARC and ME were generated from adult female mice at the proestrus or diestrus stages (Fig. S8B). DHT treatment resulted in a 2-fold increase in GnRH release compared to that in the controls during diestrus (diestrus + DHT versus diestrus + DMSO, *P* < 0.001). However, GnRH levels significantly decreased when Flut was administered alongside DHT compared to those in DHT treatment alone (diestrus + DHT + Flut versus diestrus + DHT, *P* < 0.05) (Fig. S8C).

After 8 weeks of PCOS modeling, Flut was administered directly into the 3V for 14 d using drug delivery cannulas (once every other day) (Fig. 3D). The expression levels of LH serve as an indicator of the secretion and functional dynamics of hypothalamic GnRH [25]. Time-course analysis of plasma LH levels showed that the elevated GnRH levels observed in PCOS-like mice were significantly attenuated by Flut (Fig. 3E). Systematic Flut administration restored disrupted estrous cycles (Fig. 3F and Fig. S9A) and increased ovarian volume, along with the number of corpora lutea and preovulatory follicles, indicating a restoration of ovarian function (Fig. S9B to E). Furthermore, Flut administration in sham-operated mice had no discernible effect on hormone secretion or reproductive capabilities. Administration of Flut confirmed the specific involvement of the androgen/AR signaling pathway in disrupting the GnRH-tanycyte unit, and systematic Flut administration resulted in a substantial reduction in GnRH volume and a significant increase in tanycyte process volume (Fig. 3G to I). This was accompanied by elevated levels of tanycyte mRNA and protein expression near the 3V in PCOS-like mice (Fig. 3J to L), suggesting that Flut regulates both GnRH secretion and tanycyte processes in the hypothalamus.

### Specific knockdown of *AR* in tanycytes attenuates excessive LH secretion and PCOS-like symptoms

**Fig. 4.**
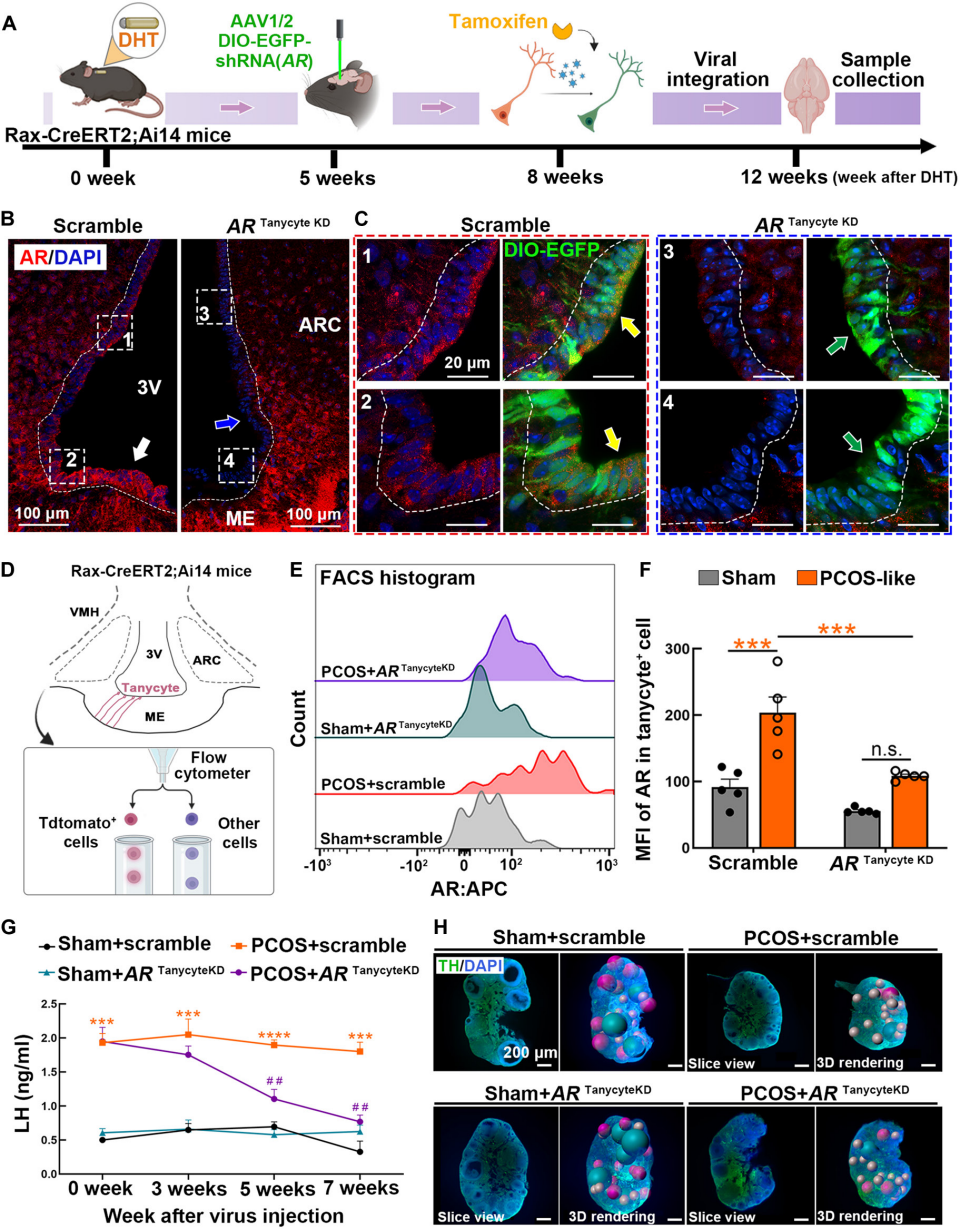
Specific knockdown of *AR* in tanycytes restored reproductive endocrine function. (A) Protocol for Cre-dependent knockdown of *AR* in tanycytes followed by the administration of DHT in Rax-CreERT2;Ai14 mice. After viral injection, mice received tamoxifen administration at 100 mg/kg per day for 5 consecutive days to induce the expression of the Cre recombinase enzyme. (B). Immunofluorescence images showing AR expression. White arrows indicate regions of AR enrichment, while blue arrows highlight reduced AR expression following tanycyte-specific knockdown. (C). High-magnification images showing AR colocalization with tanycytes. The yellow arrows indicate AR and tanycyte colabeling, and the green arrows highlight specific *AR* knockdown. (D) Tanycytes were sorted by FACS from the Rax-CreERT2;Ai14 mice injected with DIO-shAR virus. (E) FACS histogram. The vertical axis represents the number of tanycyte-positive cells. The horizontal axis represents the average fluorescence intensity of AR in tanycyte-positive cells. (F) The mean fluorescence intensity (MFI) represents the average fluorescence intensity of AR, calculated using flow cytometry data analysis software (FlowJo 9) following FACS. The quantification of AR expression levels within the sorted tanycyte population provided a reliable measure of knockdown efficiency (*n* = 5, 2-way ANOVA followed by Tukey’s post hoc test, ***P < 0.001). (G) After scramble or *AR*-specific knockdown virus injection for 0, 3, 5, and 7 weeks (DHT administration for 5, 8, 10, and 12 weeks), LH levels in peripheral blood were collected at the same time of day (8:00 AM to 10:00 AM) to characterize GnRH release (*n* = 3, 2-way ANOVA followed by Tukey’s post hoc test, ***P < 0.001 and ****P < 0.0001 versus the sham + scramble group; ^##^*P* < 0.01 versus the PCOS + scramble group). (H) Transparent and 3-dimensional reconstructed ovarian images using the iDISCO method. The Surface and Spots algorithms of the Imaris software were used to show ovarian morphology and to count the number of follicles. Corpora lutea (cyan), preovulatory follicles (red), and antral follicles (gray) are indicated.

To ascertain whether AR in tanycytes is responsible for the process changes and reproductive dysfunction triggered by androgens, we utilized the Rax-CreERT2;Ai14 conditional mouse line, which enables the cell-specific knockdown of *AR* in tanycytes. Tanycytic *AR* expression was knocked down via AAV1/2-mediated delivery of DIO-*AR*-silencing short hairpin RNA (shRNA) (*AR*^TanycyteKD^) (Fig. 4A). Immunofluorescence staining revealed that the enhanced green fluorescent protein (EGFP), transmitted by the AAV1/2 virus, extensively colocalized with areas positively stained by the tdTomato and vimentin antibodies, indicating effective viral infection of tanycytes (Fig. S10). Further immunofluorescence staining showed reduced AR expression in tanycyte cell bodies along the lateral wall of the 3V following *AR* knockdown (Fig. 4B, blue arrow). Magnified regions of interest (Fig. 4C, panels 1 to 4) provide detailed visualization: Panels 1 and 2 show colabeling of AR (red) and tanycytes (green), while panels 3 and 4 highlight a profound decrease in AR expression after *AR* knockdown. The selective *AR* knockdown was further validated by coexpression of tanycytes and AR using fluorescence-activated cell sorting (FACS) of hypothalamic tissues. TdTomato-positive cells were isolated from Rax-CreERT2;Ai14 mice injected with the AAV1/2 vector (Fig. 4D). AR expression was detected using allophycocyanin (APC)-conjugated AR antibodies (Fig. S11). In the PCOS + scramble virus group, AR was enriched in tdTomato-positive cells. Conversely, a considerable reduction in AR expression was observed in the PCOS + *AR*^TanycyteKD^ mice (Fig. 4E and F). The FACS results demonstrated that shAR virus-mediated knockdown reduced AR in tanycytes.

Consistent with previous observations, PCOS-like mice exhibited increased body weight, disrupted estrous cycles (Fig. S12A to C), elevated LH levels, and impaired ovulation (Fig. 4G and H). Compared to PCOS-like mice injected with scramble virus, *AR*^TanycyteKD^ mice exhibited significant reductions in body weight and a gradual normalization of estrous cycles. Five weeks after injection with the shAR virus, a notable decrease in LH levels was observed, which progressively returned to normal levels (Fig. 4G). Additionally, specific *AR* knockdown restored ovulatory function in PCOS-like mice, as evidenced by increased ovarian volume and elevated numbers of preovulatory follicles and corpora lutea (Fig. 4H and Fig. S12D to F). Enzyme-linked immunosorbent assay (ELISA) assays demonstrated that specific knockdown of *AR* in tanycytes reduced the pulsatility of LH release following DHT injection, underscoring the role of AR on tanycytes in regulating GnRH secretion (Fig. S13).

### The modulation of AR on tanycyte processes is contingent upon the presence of Itgb1

**Fig. 5.**
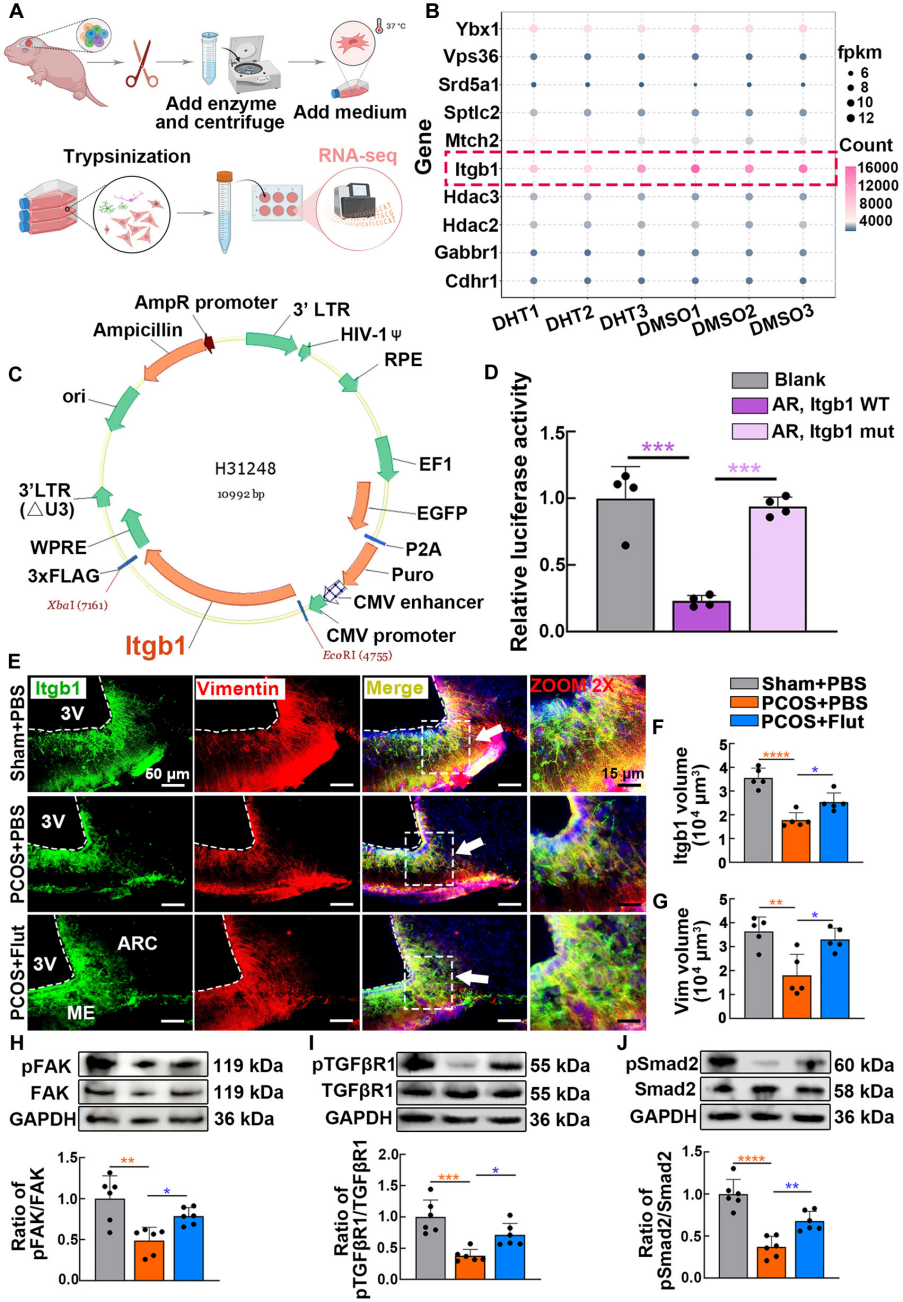
AR negatively regulates Itgb1 as a potential target gene. (A) Extraction and culture of primary tanycytes from the hypothalamus of 10-d-old rats. Primary cultured tanycytes were exposed to DHT (10 nM) for 48 h, and the total mRNA was extracted for transcriptome sequencing. (B) Fragments per kilobase million (fpkm) and count values of candidate target genes in each sample. (C) Construction of the *Itgb1* plasmid. (D) Dual-luciferase reporter assay indicated the suppressing effect of AR on Itgb1 expression. Blank plasmids were transfected as negative controls. Both wild-type and mutated *Itgb1* promoters were cotransfected with *AR* overexpression plasmids to delineate the specificity of AR’s suppression (*n* = 4, one-way ANOVA followed by Tukey’s post hoc test, ***P < 0.001). (E) Representative immunofluorescence images showing Itgb1 (green), vimentin (red), and merged channels in the ARC and ME regions of the hypothalamus from the sham + PBS, PCOS + PBS, and PCOS + Flut groups. Arrows indicate the colocalization of Itgb1 and vimentin. (F and G) Quantification of Itgb1 and vimentin volumes showed a significant reduction in the PCOS + PBS group compared to sham + PBS, with partial restoration in the PCOS + Flut group (*n* = 5, one-way ANOVA followed by Tukey’s post hoc test, *P < 0.05, **P < 0.01, and ****P < 0.0001). (H to J) Representative immunoblot bands and quantitative analysis of the expression levels of both the total and phosphorylated forms of FAK, TGF-βR1, and Smad2 (*n* = 6, one-way ANOVA followed by Tukey’s post hoc test, *P < 0.05, **P < 0.01, ***P < 0.001, and ****P < 0.0001).

To elucidate the possible mechanisms by which AR activation regulates tanycyte processes, potential targets were screened in primary cultured tanycytes. Due to the limited volume of the mouse hypothalamus, primary tanycytes were derived from the rat hypothalamus (Fig. 5A) and specific cell types were subsequently identified (Fig. S14). Transcriptome sequencing was performed on primary tanycytes exposed to androgens for 48 h. The Gene Ontology (GO) enrichment analysis revealed that the differentially expressed genes (DEGs) were notably enriched in processes related to cell developmental growth, morphogenesis, extracellular matrix binding, negative regulation of cell growth, and dynein axonemal particles (Fig. S15A). Kyoto Encyclopedia of Genes and Genomes (KEGG) pathway analysis demonstrated that the DEGs were predominantly enriched in pathways associated with the cell cytoskeleton and morphogenesis, such as regulation of the actin cytoskeleton and adherens junctions. Notably, KEGG enrichment analysis indicated a potential association between the DEGs and the regulation of GnRH secretion (Fig. S15B). In a previous study, transcriptome sequencing results from brain organoids suggested that androgen administration triggered multiple gene expression changes associated with cell proliferation [26]. By comparing these findings, we identified 51 genes that exhibited a consistent pattern with our observations. We utilized the chEA3 transcription factor prediction database to identify potential downstream targets of AR among the 51 candidate genes. This analysis revealed 15 genes as potential AR regulatory targets. Further investigation into the functions of these genes highlighted *Itgb1* as a promising AR target, suggesting a key role in regulating the morphology and function of the GnRH-tanycyte unit. *Itgb1*, a crucial regulator of cell adhesion, migration, growth, and survival, showed noticeably decreased expression following androgen treatment (Fig. 5B). To validate the interaction between AR and Itgb1, a dual-luciferase reporter system was employed. Transfection of HEK-293T cells with a plasmid overexpressing *AR* resulted in significant down-regulation of luciferase activity linked to the wild-type *Itgb1* promoter. In contrast, cells transfected with an empty plasmid (control group) showed no change in luciferase activity. Additionally, luciferase activity from the *Itgb1* promoter was no longer affected by the *AR*-overexpressing plasmid after introduction of a site-specific mutation in *Itgb1* (Fig. 5C and D)*.* The dual-luciferase assay revealed that plasmids overexpressing *AR* could inhibit the fluorescence driven by the *Itgb1* promoter, suggesting that AR may function as a transcription factor, potentially suppressing the transcription and translation of *Itgb1*.

To investigate the role of the AR/Itgb1 pathway in the androgen-induced morphological plasticity of the tanycyte cytoskeleton, primary tanycytes were cultured in vitro. These tanycytes exhibited increased AR expression and reduced Itgb1 mean fluorescence intensity upon exposure to androgens. Flut treatment up-regulated the fluorescence intensity of Itgb1 and effectively restored the normal architecture of the tanycyte cytoskeleton (Fig. S16A to D). Consistent with the immunostaining results, Flut reversed the reduction in Itgb1 caused by androgen exposure at both the mRNA and protein levels (Fig. S16E to G). Scratch wound assays indicated that androgen exposure impaired the extension and migration capabilities of tanycyte processes. Flut treatment ameliorated this damage, restoring tanycyte extension function. Notably, Flut administration in the absence of androgens did not alter the basal cellular condition, indicating its specific role in counteracting androgen-induced effects (Fig. S17A and B). Our research found that androgen exposure resulted in a significant down-regulation of FAK phosphorylation, and the attenuation of FAK activity led to a subsequent decrease in the phosphorylation of TGF-βR1 and Smad2. Notably, treatment with the anti-androgen Flut effectively restored the phosphorylation status of the FAK/TGF-βR1/Smad2 signaling axis disrupted by androgen exposure (Fig. S17C to E). Consistent with the in vitro experimental findings, Itgb1 exhibited widespread distribution around the 3V and colocalized with tanycytes in the sham + PBS group, indicating its role in maintaining tanycyte structure and function (Fig. 5E). In the PCOS + PBS mice, excessive androgen exposure resulted in a marked reduction in both Itgb1 levels and tanycyte process volumes (Fig. 5F and G). Notably, the AR antagonist Flut effectively reversed these changes, restoring both Itgb1 expression and tanycyte process volumes to levels comparable with those in the sham + PBS group. Flut treatment also restored the activity of the FAK/TGF-βR1/Smad2 signaling pathway, as indicated by the increased phosphorylation status in the PCOS + Flut group (Fig. 5H to J). These findings highlight the critical role of Itgb1 in mediating androgen-induced tanycyte dysfunction.

### Overexpression of *Itgb1* restores androgen-induced abnormal tanycytic cytoskeleton morphology

**Fig. 6.**
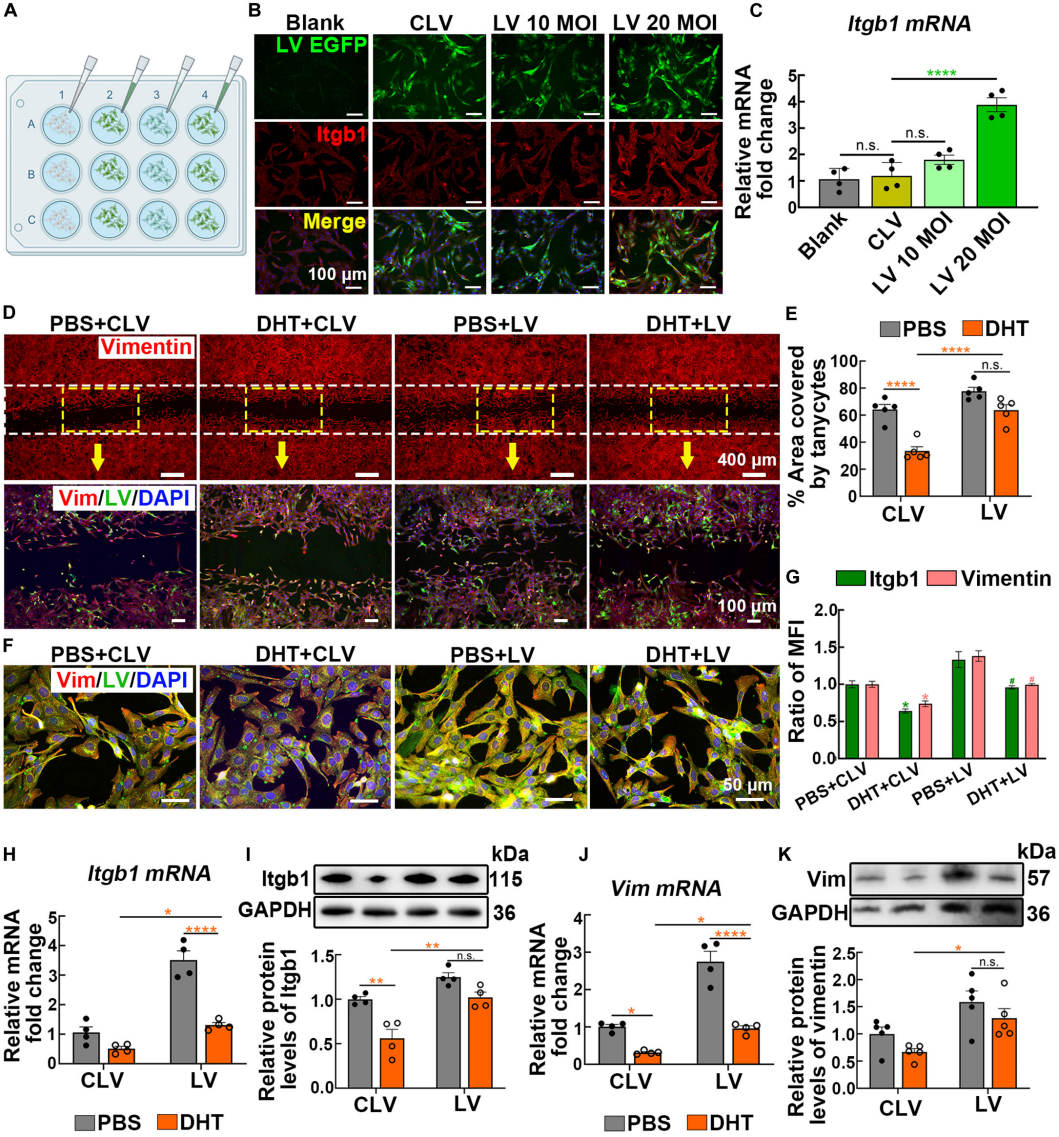
Elevated Itgb1 expression alleviated the damage caused by excessive androgen. (A) LV was designed to overexpress *Itgb1* and was specifically transfected into primary cultured tanycytes. (B) Representative immunofluorescence images of LV reporter fluorescence (EGFP, green) and Itgb1 antibody staining (red) and real-time PCR analysis (C) of *Itgb1* mRNA in each group with various dilutions of LV. Polybrene instead of LV was added to the blank group (*n* = 4, one-way ANOVA followed by Tukey’s post hoc test, ****P < 0.0001). (D) Representative images and the area covered by tanycytes (E) in a scratch wound healing assay performed on primary tanycytes. Control or *Itgb1*-overexpressing LVs were transfected into primary tanycytes that had been previously exposed to DMSO or DHT (*n* = 5, 2-way ANOVA followed by Tukey’s post hoc test, ****P < 0.0001). (F and G) Images and MFI ratios of immunolabeling for Itgb1 (green), vimentin (red), and nuclei (blue) in primary tanycytes with different treatments (*n* = 3, 2-way ANOVA followed by Tukey’s post hoc test, *P < 0.05 versus the DMSO + CLV group; ^#^*P* < 0.05 versus the DHT + CLV group). (H and I) The mRNA and protein levels of Itgb1 were evaluated using real-time PCR and WB in primary tanycytes (*n* = 4, 2-way ANOVA followed by Tukey’s post hoc test, *P < 0.05, **P < 0.01, and ****P < 0.0001). (J and K) mRNA and protein levels of vimentin in primary tanycytes (*n* = 4, 2-way ANOVA followed by Tukey’s post hoc test, *P < 0.05 and ****P < 0.0001).

Considering the inhibitory effect of AR on Itgb1 expression and tanycyte cytoskeletal integrity, we investigated whether rescuing Itgb1 expression could effectively mitigate this impairment. Lentiviruses (LVs) containing the *Itgb1* cDNA sequence along with EGFP were transfected into primary cultured tanycytes at varying dilutions (Fig. 6A). Both immunofluorescence and PCR analysis demonstrated that the virus with a multiplicity of infection (MOI) of 20 successfully elevated Itgb1 expression levels (Fig. 6B and C). Subsequently, LVs engineered to overexpress *Itgb1* were administered to primary cultured tanycytes exposed to androgen. Scratch wound assays demonstrated that *Itgb1* overexpression restored the expansion of tanycyte processes impaired by androgen (Fig. 6D and E). Primary tanycyte cultures in the DMSO + LV group showed enhanced fluorescence intensity for Itgb1 and vimentin, along with more pronounced morphological and cytoskeletal remodeling, compared with that in the control LV (DMSO + CLV) group. Crucially, the distortion observed in the tanycyte cytoskeleton due to androgen exposure was partially reversed by LVs engineered to overexpress *Itgb1* (Fig. 6F and G). In agreement with this, LV administration mitigated the reduction in *Itgb1* mRNA and protein levels induced by androgen (Fig. 6H and I). Vimentin expression was up-regulated at both the genetic and protein levels (Fig. 6J and K). Overexpression of *Itgb1* effectively mitigated androgen-induced damage to tanycytes, underscoring the pivotal role of Itgb1 in maintaining the structural integrity of tanycytes.

### Transgenic overexpression of *Itgb1* in tanycytes ameliorates reproductive endocrine disorders in mice with PCOS-like symptoms

**Fig. 7.**
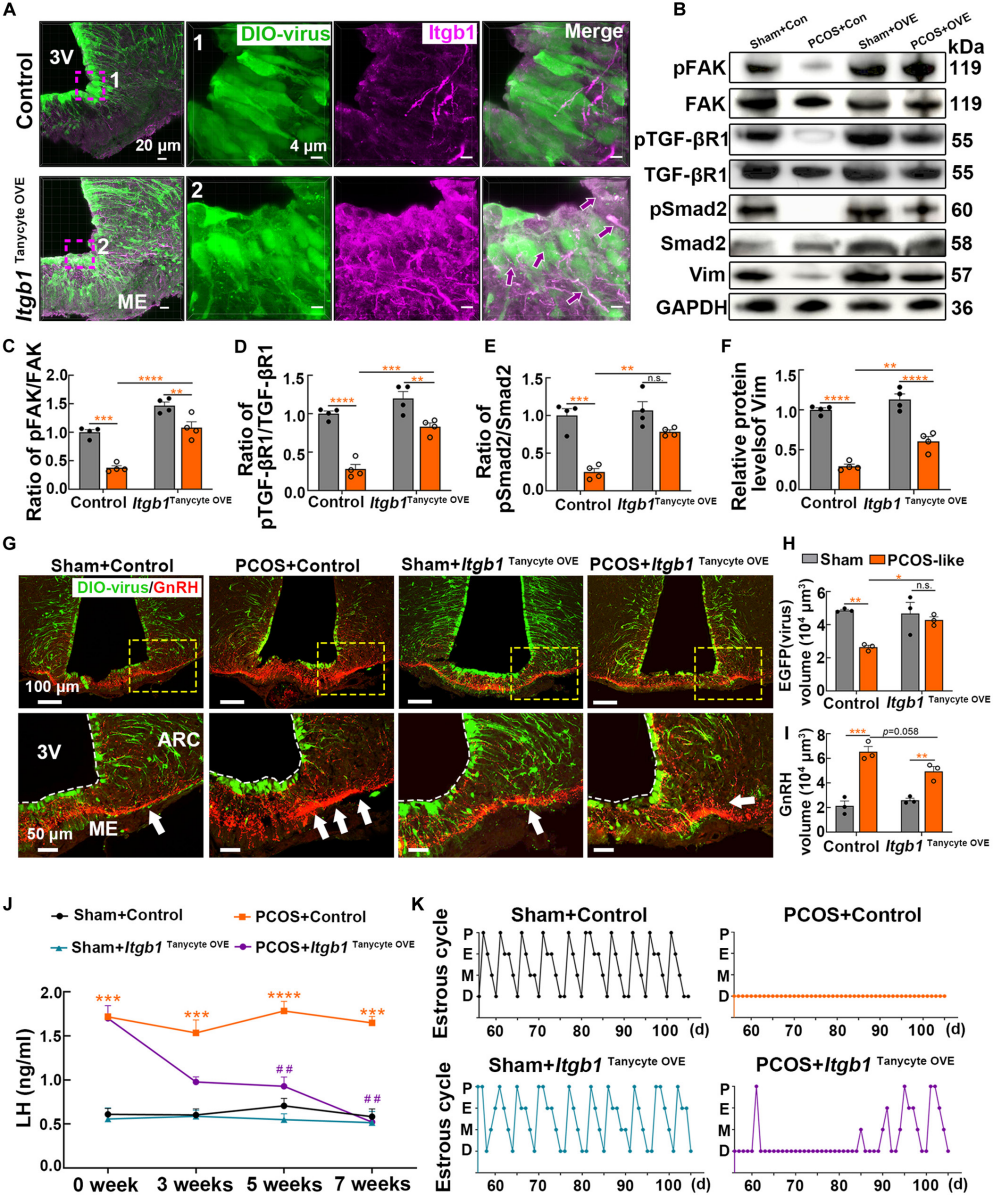
Overexpression (OVE) of *Itgb1* in tanycytes rescued the abnormal hormone release and reproductive dysfunction in PCOS-like mice. (A) Representative images showing tanycytes and Itgb1 in the ARC and ME. Tanycyte cell bodies and processes were infected with the DIO-EGFP virus, colabeled with Itgb1 staining (magenta). (B to F) Representative immunoblots and quantitative analysis of FAK, TGF-βR1, Smad2, and vimentin protein after the injection of either control or AAV1/2 *Itgb1*-overexpressing virus (*n* = 4, 2-way ANOVA followed by Tukey’s post hoc test, **P < 0.01, *** *P* < 0.001, and ****P < 0.0001). (G) Representative immunofluorescence images of DIO-virus (EGFP, green) and GnRH (red) in the hypothalamic ARC and ME regions. The arrow indicates areas of substantial changes in GnRH expression. (H and I) Quantification of the EGFP and GnRH volume in the ARC and ME (*n* = 3, 2-way ANOVA followed by Tukey’s post hoc test, *P < 0.05, **P < 0.01, and ***P < 0.001). (J) The LH expression levels in the tail-tip blood samples were measured at 0, 3, 5, and 7 weeks following virus injection using a highly sensitive LH assay kit (*n* = 3, 2-way ANOVA followed by Tukey’s post hoc test, ***P < 0.001, ****P < 0.0001 versus the sham + scramble group; ^##^*P* < 0.01 versus the PCOS + scramble group). (K) The estrus cycle of each group of mice was tested daily for 50 d (*n* = 3).

To validate our findings regarding Itgb1 in a living organism, we specifically overexpressed *Itgb1* in tanycytes by injecting the AAV1/2 DIO-*Itgb1*-EGFP vector into the lateral ventricle of Rax-CreERT2 mice [*Itgb1*^Tanycyte overexpression (OVE)^ mice], following the method described previously. Immunofluorescence results showed that the AAV1/2 virus infected both tanycyte cell bodies and processes (Fig. S18), and Itgb1 expression was elevated in *Itgb1*^TanycyteOVE^ mice compared with that in control virus-infected mice (Fig. 7A). The phosphorylation levels of proteins within the FAK/TGF-βR1/Smad2 signaling pathway were significantly down-regulated in PCOS-like mice. Intriguingly, targeted overexpression of *Itgb1* in tanycytes effectively restored the phosphorylation levels of related proteins. Activation of the FAK/TGF-βR1/Smad2 signaling pathway subsequently ameliorated the abnormalities observed in tanycytes, as evidenced by a remarkable increase in both protein and mRNA expression levels in tanycytes (Fig. 7B to F and Fig. S19A).

Moreover, we observed that the targeted overexpression of *Itgb1* effectively promoted the extension of tanycyte processes and inhibited GnRH expression near the basal capillary network (Fig. 7G). Quantitative analysis confirmed that *Itgb1* overexpression down-regulated the GnRH volumes in PCOS-like mice, suggesting its critical role in regulating GnRH-tanycyte interactions in the hypothalamus (Fig. 7H and I). Regarding peripheral phenotypes, tanycyte-specific overexpression of *Itgb1* significantly decreased body weight (Fig. S19B). Furthermore, LH levels in peripheral blood decreased gradually 3 weeks after injection of the *Itgb1*^TanycyteOVE^ virus (Fig. 7J), leading to the normalization of the estrous cycle and restoration of ovulatory function (Fig. 7K and Fig. S20). Collectively, these findings indicate that overexpression of *Itgb1* in tanycytes attenuates androgen-induced tanycyte process abnormalities by modulating the FAK/TGF-βR1/Smad2 signaling pathway.

## Discussion

In this study, a dysregulated GnRH-tanycyte unit was observed in PCOS-like mice, along with abnormal tanycyte processes in response to androgen exposure. These abnormalities may contribute to excessive GnRH secretion in PCOS. Specific AR and Itgb1 regulation in transgenic mice has provided unique insights into hypothalamic neuroglial homeostasis abnormalities associated with PCOS.

GnRH neurons are pivotal in controlling neuroendocrine function [27,28], yet their widespread distribution and unique anatomical features within the central nervous system pose challenges for targeted therapies. In PCOS patients, elevated GnRH concentrations near the ME capillary network exaggerate LH production, leading to hyperandrogenemia. However, the mechanisms underlying these alterations remain unclear [29,30]. In our study, PCOS-like mice exhibited an increased number of GnRH axonal terminals, thus providing a comprehensive perspective on GnRH distribution in this model. Importantly, intermittent delivery of the GnRH antagonist cetrorelix corrected elevated LH levels and abnormal follicular development, consistent with prior research [25]. Given the heterogeneity of PCOS, the DHT-induced model revealed that excessive GnRH secretion plays a pivotal role in exacerbating PCOS progression.

Hypothalamic GnRH release is influenced by multiple factors, including neurotransmitters such as γ-aminobutyric acid (GABA) [31,32], glutamate [3], and kisspeptins [33]. Additionally, due to their anatomical positioning and distinctive structures, glial cells have emerged as important contributors to GnRH secretion [34]. Yao and colleagues [35] highlighted the effect of glial polarization in the ARC and ME on regulating GnRH neuronal activity and DHT-induced PCOS-like traits. Tanycytes are located in the ventral portion of the 3V and serve as sensors of sex steroid activity within the neuroendocrine system [36,37]. These cells extend elongated processes to enclose axon terminals containing hormones, while their endfeet wrap around capillaries in the ME, thus solidifying their role as regulators of hormone release into the circulatory system [38]. The interaction between tanycytes and GnRH neurons is characterized by complex morphological changes and molecular pathways, involving adhesion molecules, neuronal signal molecule 7A, and PlexinC1 [7,13]. Research on the configuration and interactions between tanycytes and GnRH axon terminals primarily utilizes electron microscopy and 2-dimensional slicing techniques. However, due to the restricted volume of the hypothalamus and constraints in imaging technology, achieving high-resolution imaging and obtaining quantitative data on tanycytes and GnRH remain challenging. Using the cMAP technique and high-resolution confocal microscopy, we successfully enlarged and visualized the hypothalamus, revealing tanycyte processes closely enfolding GnRH neuron axons, resembling vines enclosing branches. Integrating tissue-clearing and expansion technology with light sheet microscopy facilitates enhanced observations at cellular and synaptic levels, thus enriching our understanding of the intricate GnRH-tanycyte unit under both physiological and pathological conditions. Further research is required to explore the molecular observations at the subcellular level and the interactions between neuronal axons and glial cells.

Tanycytes have been found to undergo periodic cytoskeletal plasticity, enabling them to directly access GnRH nerve terminals to the vessel endothelium at various reproductive stages [9]. During periods of high GnRH requirement, the tanycyte processes retract to create more extravascular space for GnRH secretion. Using a pathological PCOS mouse model [39], we visualized microscale alterations in tanycyte processes and GnRH neuronal axons within the ARC and ME of Rax-CreERT2;Ai14 mice. By leveraging the capabilities of the Imaris software for image processing, we performed 3-dimensional reconstructions and precisely quantified the volumes of the processes and axons. The contraction of tanycyte processes reduces the encapsulation and confinement of axonal terminals in GnRH neurons, potentially leading to an excessive release of GnRH. This pattern highlights the importance of tanycyte morphological plasticity in excessive secretion of GnRH. Treatment with the GnRH antagonist cetrorelix effectively mitigated PCOS symptoms; however, it did not alter the tanycyte processes. This indicates that GnRH secretion does not precede tanycyte morphology regulation. Our findings support a PCOS model in which primary pathological morphological changes occur within tanycytes, which subsequently impact the dynamics of GnRH secretion. Additionally, tanycytes are capable of secreting specific factors that directly influence the activity of GnRH neurons, including TGF-β, fibroblast growth factor 8 (FGF8), and estrogen [40–42]. Depending on the prevailing physiological conditions, these factors may either stimulate or inhibit GnRH neurons. Further investigation is essential to fully understand the complex interactions between tanycytes and GnRH neurons in patients with PCOS.

Recent evidence has indicated that tanycytes serve as biosensors for rapid responses to sex hormones [43,44]. Hormonal cues such as leptin, glucose, and insulin trigger alterations in tanycyte function. Excessive androgen is a typical symptom of PCOS [45]. Our previous study demonstrated that female rats exposed to androgens consistently exhibited increased GnRH expression [15], underscoring the pivotal role of androgens in precipitating PCOS onset. Our findings indicate that elevated androgen concentrations within the 3V can swiftly initiate the activation and subsequent atrophy of tanycyte processes and increase GnRH-dependent LH levels. In vitro experiments have shown that androgen exposure induces actin cytoskeletal remodeling in primary cultured tanycytes. Our study underscores the notable link between abnormal tanycyte processes and elevated androgen levels in PCOS-like mice, indicating an intricate interplay between tanycyte functionality and hormonal imbalances in PCOS.

To explore the potential mechanisms underlying the profound impact of androgens on tanycytes, we focused on AR, the classic receptor for androgens. Accumulating evidence highlights the novel functions of AR in the central nervous system, including the regulation of glial cell morphology and function, modulation of neurotransmitter transmission, and contribution to neural cell repair [46,47]. Here, our results revealed up-regulated hypothalamic AR in PCOS-like mice, accompanied by shortening of tanycyte processes and increased proximity of GnRH neuron terminals. Following the separation of tanycytes from the hypothalamus of mice via flow cytometry, we observed an elevated AR expression ratio in the tanycytes of PCOS-like mice compared to that in other cell types. This observation suggests that tanycyte AR may exhibit heightened sensitivity to androgenic stimuli in the context of PCOS. Acknowledging the potential for nonspecific effects of Flut injection at the 3V, we utilized Rax-CreERT2;Ai14 transgenic mice to perform targeted knockdown of *AR* in tanycytes. This precise genetic intervention successfully alleviated reproductive and endocrine disturbances induced by excess DHT, highlighting the pivotal role of tanycytic AR in the etiology of PCOS. Through a combination of in vivo and ex vivo experiments, we delineated a critical role for the AR in maintaining equilibrium within the GnRH-tanycyte unit, predominantly by modulating cytoskeletal dynamics in tanycytes.

Notably, AR expression is not exclusive to tanycytes. Additional neuronal populations within the ARC also express AR. These neurons are posited to exert distal regulatory effects on GnRH neuronal cell bodies in the MPOA region, potentially influencing neural activity and endocrine functionality [48,49]. However, numerous factors influence the release of hormones into the bloodstream [34]. We examined whether there is an additional, possibly concurrent pathway involving tanycytic modulation that might also contribute to the observed hormonal changes. The in vitro experiment excluded neurons, thus highlighting the specificity and focus of our study. By demonstrating that AR is expressed in tanycytes and linking this expression to functional outcomes (such as altered GnRH/LH secretion), our study provides valuable insights into PCOS clinical interventions by showing that inhibiting AR activity may rectify functional abnormalities in the GnRH-tanycyte unit. Strategies to accomplish this entail the development of central antagonists specific to tanycytic AR, receptor fusion proteins, or monoclonal antibodies targeting tanycytic AR. Further research is warranted to explore the interplay between neuronal and tanycytic AR signaling pathways. We propose more comprehensive studies using dual Cre lines that would allow the simultaneous knockdown of AR in tanycytes and neurons to elucidate their respective and relative contributions to PCOS pathology fully.

AR-mediated signaling is hypothesized to regulate the morphological plasticity of glial cells. Functional enrichment analysis and target gene prediction derived from transcriptome sequencing data suggested that *Itgb1* may function as a target gene for AR and affect modulating the plasticity of tanycyte processes. Itgb1 functions as a crucial cell adhesion molecule and is pivotal in various cellular processes, including cell proliferation, cytoskeletal remodeling, gene expression, and differentiation [50,51]. Selective knockout of *Itgb1* in tanycytes results in alterations in the estrous cycle accompanied by elevated circulating levels of LH [9]. A previous study showed that Itgb1 is negatively regulated by androgens, which influence the fate of spermatogonia progenitors [52]. Our analysis revealed a causal interaction between AR and Itgb1 in PCOS-like mice. AR is up-regulated and subsequently binds to and inhibits *Itgb1* transcription, further inhibiting the downstream FAK/TGF-βR1/Smad2 signaling pathway. This discovery highlights the critical role of the FAK/TGF-βR1/Smad2 pathway in mediating the effects of Itgb1 on tanycyte plasticity. Suppression of Itgb1 by AR disrupts this signaling cascade, which is essential for maintaining normal cell morphology and function. Future studies should focus on elucidating the precise role of this pathway in tanycytes and explore how its dysregulation contributes to the pathogenesis of PCOS.

By employing an LV to overexpress *Itgb1* specifically in tanycytes, we demonstrated that Itgb1 contributes to maintaining tanycyte morphology under androgen exposure. This therapeutic effect is closely associated with the reactivation of the FAK/TGF-βR1/Smad2 signaling pathway. Moreover, the specific overexpression of *Itgb1* in tanycytes effectively reversed endocrine dysfunction in PCOS-like mice, highlighting the precision and efficacy of Itgb1-based interventions. Our research unifies these elements into a coherent mechanism whereby the elevated AR in PCOS-like mice impedes the function of Itgb1 and the FAK/TGF-βR1/Smad2 signaling pathway, which is responsible for maintaining the normal morphology and cytoskeleton of tanycytes. The administration of AR antagonists or the AAV1/2-DIO-*Itgb1* virus results in restoring Itgb1 expression and the FAK/TGF-βR1/Smad2 signaling pathway. This restoration, in turn, helps restore tanycyte morphology and thus maintains a normal GnRH-releasing environment. By knocking down *AR* and overexpressing *Itgb1* in tanycytes, we investigated the crucial role and molecular mechanisms by which the androgen/AR/Itgb1 pathway mediates the cytoskeletal morphology of tanycytes in PCOS. This approach helped identify potential therapeutic targets and enhanced our understanding of the broader neuroendocrine dysfunctions associated with PCOS. This might lead to more effective treatments than currently available, focusing solely on symptom management.

**Fig. 8.**
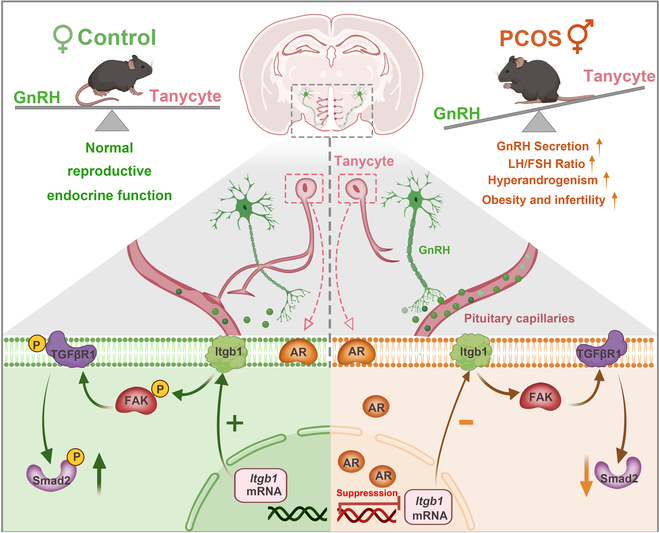
Working principle of the GnRH-tanycyte unit. During the normal reproductive metabolic cycle, tanycyte processes enclose GnRH neuron axon terminals, creating a diffusion barrier for GnRH release. However, excessive androgen impairs the physical sheaths that envelop GnRH-containing neuron terminals and creates increased extravascular space for GnRH secretion in PCOS.

Our study concentrated on the alterations in endocrine function resulting from neuroglial plasticity, deepening our understanding of the role played by the GnRH-tanycyte unit. Tanycytes, like vines, orchestrate the release of GnRH into the portal vasculature, a process contingent on their capacity to adjust their morphology in response to elevated androgen levels. We demonstrated that the AR, expressed in tanycytes, plays a dual role in the retraction of GnRH terminals and in promoting their ensheathment by tanycytic endfeet via Itgb1 (Fig. 8). Further detailed mechanistic and epidemiological investigations are required to determine the role of tanycytes in the occurrence and development of PCOS.

## Materials and Methods

### Animals

All C57BL/6J mice and Wistar rats were purchased from the Slack Laboratory Animal Center (Shanghai, China). *tdTomato*^loxP−STOP-loxP^, C57BL/6J background (also known as Ai14, RRID: IMSR_JAX:007914), were generously provided by S. Liu (Fudan university, China). The GnRH neuron reporter mouse line was engineered by Shanghai Biomodel Organism Science & Technology Development Co. Ltd. We generated *tdTomato*^GnRH^ mice, which express tdTomato red fluorescence signal in GnRH neurons, including their projections, by crossing GnRH-CreERT2 mice with those allowing for Cre-dependent expression of tdTomato from the ROSA26 locus. The Rax-CreERT2;Ai14 mouse line was graciously provided by Q. Wu [53] (Chinese Academy of Sciences, Beijing). The animals were maintained through backcrossing in a C57BL/6J genetic background. Transgenic model mice were bred and genotyped in house to generate experimental animals. The animals were housed in a temperature-controlled room (21 to 22 °C) with a 12-h light/dark cycle and ad libitum access to food and water. All experiments were conducted in accordance with the National Institutes of Health Guide for the Care and Use of Laboratory Animals (NIH Publication No. 8023, revised 1978) and were approved by the Animal Care and Use Research Ethical Standards of Fudan University (No. 20210302-086, Shanghai, China).

### Tissue-clearing method: iDISCO

Sample clearing was conducted following the iDISCO protocol as described previously [54]. For whole-brain clearing, paraformaldehyde (PFA)-fixed samples were washed twice with 0.01 M PBS for 1 h and then dehydrated in a series of increasing concentrations of methanol (20%, 40%, 60%, 80%, 100%) for 6 h per step at room temperature. Subsequently, the brains were bleached overnight at 4 °C in methanol containing 5% hydrogen peroxide. The dehydrated and bleached brains were then incubated with a solution of 66% dichloromethane (catalog no. 34856, Sigma-Aldrich) in methanol for 6 h until they sank. The rehydrated samples were incubated overnight at 37 °C in a blocking solution composed of 10% normal donkey serum and 10% dimethyl sulfoxide (DMSO). The following day, the brains were incubated with GnRH primary antibody (1:100 dilution, catalog no. G8294, Sigma-Aldrich) for 7 d at 37 °C on a swivel platform. The samples were then incubated with the secondary antibody (donkey anti-rabbit Alexa 647, Invitrogen) for 7 d. After completing the antibody incubation, the samples underwent dehydration once more. For clearing and staining [tyrosine hydroxylase, 1:50 dilution, catalog no. ab112, Abcam; 4′,6-diamidino-2-phenylindole (DAPI), 1:100 dilution, catalog no. 62248, Thermo Fisher Scientific] of the ovaries, the processing time was halved. The samples were then cleared and stored in dibenzyl ether (catalog no. 33630, Sigma-Aldrich) until imaging. The transparent blocks were imaged using a light sheet microscope (LS18, Nuohai Life Science) with semi-automatic calibration capability. The brain images were analyzed using Imaris software (v9.8, Andor Technology, Britain) for stitching and 3-dimensional reconstruction.

### BIRDS for mouse brain mapping

The original 3-dimensional brain dataset was preprocessed to adjust the slice order and orientation and thus align it with the Allen Brain Atlas. The BIRDS (bi-channel image registration and deep-learning segmentation) plug-in in ImageJ was launched. Once the save path was set, the 3-dimensional brain dataset to be analyzed was selected, making sure to adjust any relevant parameters within the plug-in for optimal processing and visualization of the dataset [55]. Seven cross-sectional images were selected and aligned with their corresponding images in the Allen Brain Atlas, followed by downsampling for further analysis. Brain regions within the whole-brain image were segmented using the Marching Cubes algorithm, referencing the Allen Brain Atlas. Key cross-sections from 3 orthogonal planes (*X*, *Y*, and *Z*) were identified to establish a standard baseline. ImageJ was then integrated with Imaris software to create various surfaces mapped to the Allen Brain Atlas within the 3-dimensional brain data, thus providing a precise structural delineation for analysis.

### Tissue-clearing method: cMAP

The PFA-fixed brain samples were washed 3 times with 0.01 M PBS and then incubated in 50 ml of solution 1 (S1; fast delipidating solution 1) containing urea (150 g), *N*-butyl diethanolamine (100 g), and Triton X-100 (100 g) dissolved in ddH_2_O (650 g). The samples were placed on a swivel platform at 37 °C, and the S1 solution was replaced every 24 h until the sample become transparent. The brains were then washed 3 times with PBS to remove residual S1. To evaluate the morphological changes in tanycytes and GnRH, the hypothalamus was stained with chicken anti-vimentin (1:50 dilution, catalog no. AB5733, Millipore) and rabbit anti-GnRH (1:100 dilution, catalog no. G8294, Sigma-Aldrich) primary antibodies for 3 d at 37 °C. Next, the stained tissues were incubated with goat anti-chicken conjugated with Alexa Fluor 488 and donkey anti-rabbit conjugated with Alexa Fluor 647, respectively. The samples were immersed in 10 ml of gel monomer solution [prepared with acrylamide, sodium acrylate, and 2′-azabis (2-imidazoline) dihydrochloride] and incubated at 4 °C for 48 h. Polymerization of the monomer solution was initiated by ultraviolet irradiation. The polymerized rubber block was then submerged in ddH_2_O (500 ml), with the water being replaced every 24 h. Three-dimensional imaging was performed when the rubber block swelled to 3 times its original volume. For imaging, the expanded brain samples were placed in an imaging chamber filled with ddH_2_O.

### Establishment of the PCOS model

We utilized the DHT (catalog no. A8380, Sigma-Aldrich)-induced PCOS mouse model, which has been extensively documented and validated in prior research [56,57]. This model is valued for its reproducibility and relevance to human PCOS, especially in terms of mimicking the hyperandrogenic environment characteristic of the condition. Mice in the PCOS group were implanted with subcutaneous sustained-release soft silastic capsules containing 15 mg of DHT at 3 weeks of age. The DHT concentration in each implant was carefully calibrated to maintain levels that effectively simulate the hyperandrogenic environment characteristic of PCOS. A small incision was made in the nape of the neck after the area was disinfected with iodine and 70% ethanol. The implant was then inserted, and the incision was sealed with surgical sutures. The stage of the estrous cycle was determined by observing the cellular morphology and cell type in vaginal smears. The model was deemed successful if the mice exhibited prolonged diestrus phases, an indicator of disrupted estrous cyclicity typical in PCOS. Prior to initiating any experimental procedures or sampling, vaginal cytology was performed to confirm that the mice were in the diestrus phase, thereby eliminating any morphological or hormonal variations attributable to differing cycle stages.

### Enzyme-linked immunosorbent assay

To examine the regulatory effects of DHT on LH release, tail-blood samples (5 μl) were collected at 10-min intervals over a 2-h period following the administration of DHT. In a second experiment, tail blood was collected before Flut administration and on days 2, 7, and 14 of each treatment at the same time each day (8:00 AM to 10:00 AM). The blood samples were diluted in 0.01 M PBS with heparin sodium (175 IU). LH levels were detected using a mouse ELISA kit (BPE20343, Lengton Biological Technology), and serum levels of LH, follicle stimulating hormone (FSH; BPE20419), DHT (BPE20577), testosterone (T; BPE20375), and estradiol (E2; BPE20381) were measured using ELISA kits according to the manufacturer’s instructions. The inter-assay variability of 12% and intra-assay variability of 10% are based on the manufacturer’s specifications, including the coefficients of variation provided in the kit documentation. All ELISA procedures were performed in strict accordance with the manufacturer’s protocols and recommended sample processing methods. Duplicate measurements were conducted to minimize user-related variability and ensure consistency.

### Tissue processing and immunofluorescence staining

Mice in the diestrus stage were anesthetized using tribromoethanol/avertin (350 mg/kg, intraperitoneally) and subsequently transcardially perfused with PBS (60 ml) followed by 4% PFA (30 ml). The brains were coronally sliced at a thickness of 30 μm using a freezing microtome (Leica Biosystems). Free-floating sections were washed in PBS for 10 min and then blocked in QuickBlock immunostaining blocking solution (P0260, Beyotime) for 15 min. The sections were then incubated with diluted primary antibodies as follows: rabbit anti-GnRH (1:2,000 dilution, catalog no. G8294, Sigma-Aldrich), rabbit anti-c-Fos [1:100 dilution, catalog no. 31254, Cell Signaling Technology (CST)], chicken anti-vimentin (1:1,000 dilution, catalog no. AB5733, Millipore), mouse anti-AR (1:200 dilution, catalog no. sc-7305, Santa Cruz Biotechnology), rabbit anti-glial fibrillary acidic protein (GFAP) (1:200 dilution, catalog no. 80788S, CST), and rabbit anti-Itgb1 (1:500 dilution, catalog no. 12594-1-AP, Proteintech). After incubating overnight with the primary antibodies, the brain slices were incubated with donkey anti-rabbit Alexa Fluor 488, goat anti-chicken Alexa Fluor 488, and donkey anti-mouse Alexa Fluor 594 (Invitrogen), respectively, for 2 h at room temperature. The immunofluorescence results were captured using a fluorescence microscope (NCF950, Ningbo Yongxin Optics Co. Ltd.). A spatial array high-resolution confocal microscope was used to observe the tanycytic ultrastructure [Nikon Spatial Array Confocal (NSPARC)]. The acquisition of images was performed using an inverted confocal microscope, and high-magnification photomicrographs were acquired with a 60× objective combine with the ZOOM function. ImageJ-Pro Plus 6.0 (Media Cybemetics), Imaris software (v9.8, Andor Technology, Britain), NIS-Elements Viewer 5.21 (Nikon Corporation), and Photoshop CC (Adobe Systems, San Jose, CA, RRID: SCR014199) were used to quantify and merge the images.

### Quantitative real-time PCR analysis

The TRIzol method (Invitrogen, Thermo Fisher Scientific) was used for RNA isolation from brain tissue and cultured cells according to the manufacturer’s instructions. The quantity and purity of total RNA were assessed using a NanoDrop ND-1000 spectrophotometer (NanoDrop Technologies Inc.), and equal quantities of RNA (1 μg) were reverse transcribed to synthesize cDNA (catalog no. RR036A, TaKaRa). Quantitative real-time PCR (qRT-PCR) was performed using the Applied Biosystem 7500 Real-Time PCR system with a TB Green Premix Ex Taq kit (catalog no. RR420A, TaKaRa) in a total reaction volume of 20 μl. The primer sequences were as follows: *DARPP32* (mouse) forward: 5′-TCTCTGCTCTCAAGACCTGGAACTG-3′, reverse: 5′-GACCTCTGCCTCCGACACTCTC-3′; *AR* (mouse) forward: 5′-CGAGGCAGCAGCATACCAGAATC-3′, reverse: 5′-GCTTGATACGGGCGTGTGGATG-3′; *Itgb1* (mouse) forward: 5′-AACTAACCAGCATTCCACCACAGAC-3′, reverse: 5′-AGGCAGAGAGGGCATCTACCAAG-3′; *GAPDH* (mouse) forward: 5′-AAATGGTGAAGGTCGGTGTG-3′, reverse: 5′-AGGTCAATGAAGGGGTCGTT-3′; *Itgb1* (rat) forward: 5′-GGGCAGGCTTGTGGTGAGAG-3′, reverse: 5′-GGCAGGTGAGACGGCTAAGATG-3′; *DARPP32* (rat) forward: 5′-AGAGGAGGACGAAGAAGAAGACAG-3′, reverse: 5′-CCATAGCAGGACATCACAGGATTC3′; *GAPDH* (rat) forward: 5′-CAAGTTCAACGGCACAGTCAAGG-3′, reverse: 5′-CGTACTCCTGGAAGATGGTGATGG-3′. The relative amount of the target gene was calculated using the 2^−ΔΔCt^ method.

### WB analysis

Brain tissues and cultured cells were lysed in radioimmunoprecipitation assay (RIPA) buffer (catalog no. P0013B, Beyotime) containing phenylmethylsulfonyl fluoride (PMSF) (catalog no. ST506, Beyotime) and Halt Phosphatase Inhibitor Cocktail (catalog no. 78427, Thermo Fisher Scientific) at a ratio of 100:1:1. Supernatants were collected after centrifugation (12,000*g*, 20 min, 4 °C), and the protein concentrations were determined using the Bradford method (catalog no. 23225, Thermo Fisher Scientific). Protein samples were separated by 10% sodium dodecyl sulfate–polyacrylamide gel electrophoresis and subsequently transferred to polyvinylidene fluoride membranes (Millipore Sigma). The membranes were blocked with 5% (w/v) nonfat milk at room temperature for 2 h and then incubated overnight at 4 °C with primary antibodies against vimentin (1:1,000 dilution, catalog no. 5741S, CST), AR (1:1,000 dilution, catalog no. ab133273, Abcam), Itgb1 (1:1,000 dilution, catalog no. 12594-1-AP, Proteintech), FAK (phospho-Y397) (1:500 dilution, catalog no. ab81298, Abcam), FAK (1:500 dilution, catalog no. ab40794, Abcam), TGF-βR1 (phospho-S165) (1:1,000 dilution, catalog no. ab112095, Abcam), TGF-βR1 (1:1,000 dilution, catalog no. ab121024, Abcam), Smad2 (phospho-S467) (1:500 dilution, catalog no. ab280888, Abcam), and Smad2 (1:500 dilution, catalog no. ab33875, Abcam). The protein expression levels were detected using an enhanced chemiluminescence ECL kit (WBKLS0500, Millipore) with an ImageQuant LAS4000 mini-gel imaging system (GE Healthcare Life Sciences). All specific protein band densities were normalized using glyceraldehyde-3-phosphate dehydrogenase (GAPDH) or the corresponding total protein as the loading control and were analyzed using Image-Pro Plus 6.0 (Media Cybernetics).

### Stereotaxic surgery and drug administration

Mice in the diestrus stage were anesthetized and positioned in a stereotaxic apparatus for drug administration (RWD Life Science). The injection site was determined utilizing the mouse brain atlas and was located precisely at –1.7 mm posterior to the bregma and –5.5 mm ventral to the brain surface. A total of 1 μl of either PBS or DHT (10 nM) was injected into the 3V using a Hamilton syringe [58]. For Flut (HY-B0022, MedChemExpress, China) administration, cannula implantation (RWD Life Science) was performed 10 d prior. Flut was prepared at a concentration of 50 mg/ml in DMSO to create a stock solution. The drug was administered intraventricularly into the 3V at a dosage of 1 mM, which was the quantity determined from our preliminary studies to effectively inhibit AR without causing adverse effects. Sham-operated mice received identical injections of the AR antagonist. These mice served as controls to assess the basal impact of Flut on endocrine functions. Injections were given once every other day for a duration of 14 d. Control mice received equivalent volumes of the vehicle (DMSO diluted in PBS) following the same schedule in order to account for any effects due to the administration procedure itself. Blood samples were collected at 0, 2, 7, and 14 d after the initiation of treatment to measure LH levels. The estrous cycle was monitored via vaginal cytology performed daily throughout the treatment period to assess the impact of Flut on cyclicity. For GnRH antagonist administration, adult female PCOS-like mice were injected intraperitoneally with 200 μl of PBS containing cetrorelix acetate (0.5 mg/kg, every second day for 16 d) [16].

### Hypothalamus explant culture

The explants were prepared according to the protocols described previously [59,60]. Female mice (aged 3 to 4 months) with regular estrous cycles were sacrificed on the day of diestrus or proestrus. The brains were promptly harvested, and hypothalami containing the ME and ARC were microdissected under a stereomicroscope. Explants were quickly dissected and placed in a microcentrifuge tube containing D-Hank’s solution (300 μl) saturated with 95% O_2_ and 5% CO_2_. At the end of the dissections, the explants were preincubated with fresh artificial cerebrospinal fluid (CSF) medium (300 μl) with the following composition: 117 mM NaCl, 4.7 mM KCl, 1.2 mM NaH_2_PO_4_, 25 mM NaHCO_3_, 2.5 mM CaCl_2_, 1.2 mM MgCl_2_, and 10 mM glucose (pH 7.4), bubbled with 95% O_2_ and 5% CO_2_ for 10 min. After preincubation, tissues were placed in fresh artificial CSF medium (200 μl) containing either DHT (10 nM) or DHT + Flut (1 mM) for an additional 4-h incubation. The drugs were initially dissolved in DMSO (final concentration: 0.0025%). The explants were subsequently retrieved and extracted for morphological analysis. The medium was collected after drug treatment and incubated at 37 °C for 4 h with shaking at 300 rpm. The GnRH content was detected following a GnRH ELISA protocol (BPE20507, Lengton Biological Technology).

### *AR* knockdown and *Itgb1* overexpression in tanycytes

Rax-CreERT2;Ai14 mice were subjected to PCOS modeling by subcutaneously implanting DHT capsules at 3 weeks of age. The sham group underwent the same procedure but received drug-free capsules. After 5 weeks, Rax-CreERT2;Ai14 mice received stereotaxic injections of the Cre-dependent AAV1/2 virus into the lateral ventricle to achieve targeted gene knockdown or overexpression in tanycytes. For *AR*-specific knockdown, mice were injected with either AAV 1/2 rAAV-CMV-DIO-(EGFP-U6)-shRNA (*AR*)-WPRE-hGH polyA or rAAV-CMV-DIO-(EGFP-U6)-shRNA (scramble)-WPRE-hGH polyA (serotype 1:2 chimeric; titer = 5 × 10^10^, BrainVTA) in the lateral ventricle (2 μl; at 0.2 μl/min; anteroposterior, –0.3 mm; midline, –1 mm; dorsoventral, –2.5 mm). Similarly, *Itgb1* overexpression in tanycytes was achieved by injecting the AAV 1/2 rAAV-CMV-DIO-*Itgb1*-P2A-EGFP-WPRE-hGH polyA or rAAV-CMV-DIO-EGFP-WPRE-hGH polyA (serotype 1:2 chimeric; titer = 5 × 10^10^, BrainVTA) into the lateral ventricle of 8-week-old female mice. Following viral delivery, tamoxifen (MedChemExpress, HY-13757A) was administered intraperitoneally at a dose of 100 mg/kg body weight for 5 consecutive days to activate Cre recombinase and induce gene-specific knockdown or overexpression in tanycytes.

### Tanycyte isolation by FACS

The hypothalamus, including the ME and ARC, from Rax-CreERT2;Ai14 mice injected with AAV1/2 GFP-shRNA-*AR* or scramble virus were microdissected, and the tissues were enzymatically dissociated using a Papain Dissociation System (20 units/ml, catalog no. P4762, Sigma) and DNase I Solution (1 mg/ml, catalog no. 10104159001, Roche) to obtain single-cell suspensions. Diluted Percoll was used for cellular separation. FACS experiments were performed using a BD FACSVerse Cell Analyzer. Fixable Viability Stain 510 (1:1,000 dilution, catalog no. FVS510, BD Horizon) was used to discriminate viable cells from nonviable cells. The cell sorting decision was based on measurements of tdTomato fluorescence (tdTomato: excitation: 554 nm; detection: 581 nm) by screening cell suspensions from brain tissues (ME and ARC). APC-conjugated AR flow cytometry antibody (1:100 dilution, catalog no. IC5876A, R&D Systems) was used to identify AR-positive cells.

### Primary cultures of hypothalamic tanycytes

Primary tanycytes were isolated from the hypothalamus of 10-d-old Wistar rats and cultured using established methods [61,62]. The hypothalamus was carefully removed and dissected to obtain the region close to the ependymal layer. Papain (2 mg/ml, catalog no. P4762, Sigma-Aldrich) and DNase I (1 mg/ml, catalog no. 10104159001, Roche) were used to enzymatically hydrolyze the tissue block. The trypsinized tissue was subsequently transferred to culture medium consisting of Dulbecco’s modified Eagle’s medium (DMEM)/F12 (Invitrogen) supplemented with 10% (v/v) fetal bovine serum (Gibco). The cell suspension was passed through a 70-μm nylon mesh (catalog no. FSTR070, Beyotime) for filtration and subsequently centrifuged at 233*g* for 10 min. The dissociated cells were then seeded at a density of 1.2 × 10^5^ cells/cm^2^ in 75-cm^2^ culture flasks that had been pretreated with poly-l-lysine. The cells were maintained in a humid atmosphere of 5% CO_2_ and 95% air at 37 °C. After a growing period of 10 d, tanycytes were isolated from other contaminating cells though overnight shaking at 250 rpm and then plated onto 10-cm^2^ dishes for further investigation. Cell cultures from the ME were examined, and a large number of tanycytes, but only a few astrocytes, were observed.

### Transcriptome sequencing and data analysis

Total RNA was isolated from primary cultured tanycytes exposed to DHT (10 nM) for 48 h. Transcriptome sequencing and cDNA library construction were undertaken by Novo Gene Biotech Co. Ltd. (Beijing, China). To obtain high-quality reads, raw data were processed to remove adapter sequences, reads containing ambiguous bases, and low-quality reads with a base quality Phred score of ≤20. Differential expression analysis was conducted using the DESeq2 software, with the significance threshold established at log_2_ fold changes > 0.05. Furthermore, functional enrichment analysis, encompassing GO terms and KEGG pathways, was performed using the clusterProfiler package (version R4.1.3).

### Dual-luciferase reporter assay

The JASPAR database (https://jaspar.genereg.net/) was used to predict the binding sites between AR and Itgb1. Plasmids with luciferase-fused genes were constructed using conventional methods. HEK-293T cells were collected and seeded in 96-well plates at a density of 3 × 10^4^ cells per well following centrifugation and resuspension 1 d before transfection. Subsequently, a mixture of *Itgb1* promoter (0.2 μg), AR overexpression plasmid (0.5 μg), and Renilla luciferase gene reporter plasmid (0.02 μg) was transfected into the HEK-293T cells. The wild-type binding sequence in the *Itgb1* promoter that we targeted for mutation was CTGAGGGACACGGCGGGCCGAG. This sequence has undergone complementary mutations (GACTCCCTGTGCCGCCCGGCTC) in the *Itgb1* promoter. Preliminary bioinformatics analysis suggests that this sequence is a crucial regulatory element, potentially playing a crucial role in gene regulation through AR binding. By analyzing mutations in the *Itgb1* promoter and observing alterations in gene activity through luciferase assays, we could identify the role of these sites in AR-mediated gene regulation. Plasmid H352 pGL 4.10, which expresses Renilla luciferase, was used as the internal control to correct for differences in transfection efficiencies. The transfection was performed with Hieff Trans Liposomal Transfection Reagent (catalog no. 40802ES02, Yeasen Biotechnology) following the manufacturer’s protocol. The relative activity of Renilla and dual-luciferase was measured using a Duo-Lite Luciferase reporting kit (catalog no. DD1205, Vazyme) 48 h after transfection.

### Wound-healing assays of primary tanycytes

Tanycytes were seeded in 6-well plates at a high density of 2 × 10^6^ cells per well and cultured in DMEM/F-12 supplemented with 10% (v/v) fetal bovine serum. Subsequently, a sterile pipette tip was used to create a straight “scratch” with a gap measuring 300 to 400 μm across the cell monolayer. The cells were then washed with PBS twice to remove any detached cells. For further experiments, the cells were treated with either DMSO (as the control), DHT, or Flut for 48 h. Afterward, the cells were fixed in 4% PFA and permeabilized with 0.3% Triton X-100 for 1 h prior to antibody staining. The area occupied by tanycyte processes was quantified using ImageJ software.

### LV transfection

LV was used to overexpress genes in primary cultured tanycytes. The LV-mediated gene-overexpressing vector specifically targeting Itgb1 was designed, packaged, and purified by Obio Technology. The primary tanycytes were initially seeded in 6-well plates (40% confluency) and incubated in DMEM/F12 containing various dilutions of LV. After 12 h of transfection, the medium was replaced with complete medium and incubated for 72 h in a cell incubator to determine the optimal dilutions. Subsequently, 20 MOI of LV targeting Itgb1 was incubated after the primary cultured tanycytes were treated with DHT. Subsequent cell experiments were conducted once gene overexpression was completed.

### Statistical analysis

Statistical analyses were performed using GraphPad Prism Software (v 9.0). Statistical analyses commenced with tests for normality (Shapiro–Wilks test and Q–Q plots) and homogeneity of variances (Brown–Forsythe tests) to confirm the suitability of the selected statistical tests. Data are presented as the mean ± SEM. An independent sample *t* test was used when comparing 2 independent groups. One-way analysis of variance (ANOVA) and 2-way ANOVA were used for multiple group comparisons. Statistical differences between groups were considered significant for *P* < 0.05.

## Data Availability

Data used in this article are available from the authors upon request. The raw transcriptome sequencing data are available at the following link: https://bigd.big.ac.cn/gsa/browse/CRA016869.
