# Supplementary material for: Regulating Integrin β1 to Restore Gonadotropin-Releasing Hormone–Tanycyte Unit Function in Polycystic Ovary Syndrome-Related Hypothalamic Dysregulation
Source: Research (Wash D C). 2025 Feb 19;8:0619. doi: 10.34133/research.0619 (PMC11836200; doi:10.34133/research.0619)
Supplement: Supplementary 1 — Figs. S1 to S20 [file research.0619.f1.docx]

**Supporting Information**

**Regulating Itgb1 to Restore GnRH-Tanycyte Unit Function in PCOS-Related Hypothalamic Dysregulation**

Yu Wang ^1,2^, Xiaoyu Tong ^1,2^, Yan Xiao ^1,2^, Yicong Wang ^1,2^, Wei Hu ^1,2^, Wenhan Lu ^3^, Yuning Chen ^1,2^, Jiajia Li ^1,2^, Wenhao Gao ^1,2^, Hongru Gao ^1,2^, Yicheng Tian ^1,2^, Sizhe Dai ^1,2^, and Yi Feng ^1,2^*

^1^ Department of Integrative Medicine and Neurobiology, School of Basic Medical Sciences, State Key Laboratory of Medical Neurobiology and MOE Frontiers Center for Brain Science, Institutes of Brain Science, Fudan University, Shanghai, 200032, China.

^2^ Shanghai Key Laboratory of Acupuncture Mechanism and Acupoint Function, Shanghai Institute of Acupuncture and Moxibustion, Shanghai, 200433, China.

^3^ Department of Ophthalmology & Visual Science, Eye & ENT Hospital, Shanghai Medical College, Fudan University, Shanghai, China.

* Corresponding author. Department of Integrative Medicine and Neurobiology, School of Basic Medical Sciences, Fudan University, Shanghai, China.

*E-mail*: fengyi17@fudan.edu.cn (Y. Feng)

**
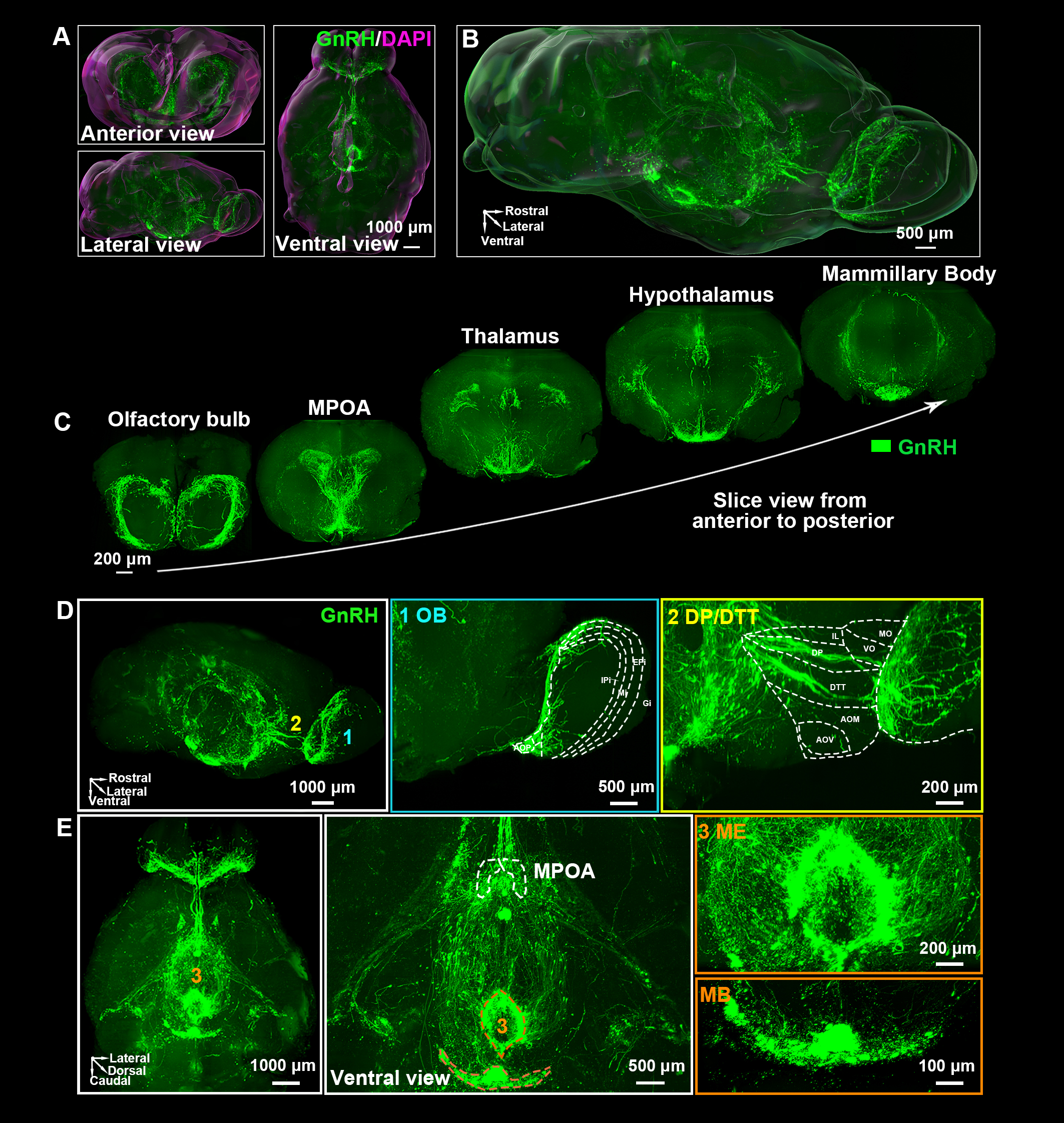
**

**Fig. S1.** Distribution of GnRH neurons in the whole mouse brain. (A and B) Adult female mouse brain cleared using iDISCO showing multiple views of GnRH (green) and DAPI (magenta) positive signals. (C) Composite images of representative GnRH neuron distribution across the mouse brain. GnRH were mainly detected in the olfactory bulb, MPOA, thalamus, hypothalamus, and mammillary body. (D) Lateral view of GnRH neurons in the OB and DP/DTT. (E) Ventral view of GnRH in the hypothalamus. Densely fluorescent signals were distributed in the ME and MB.


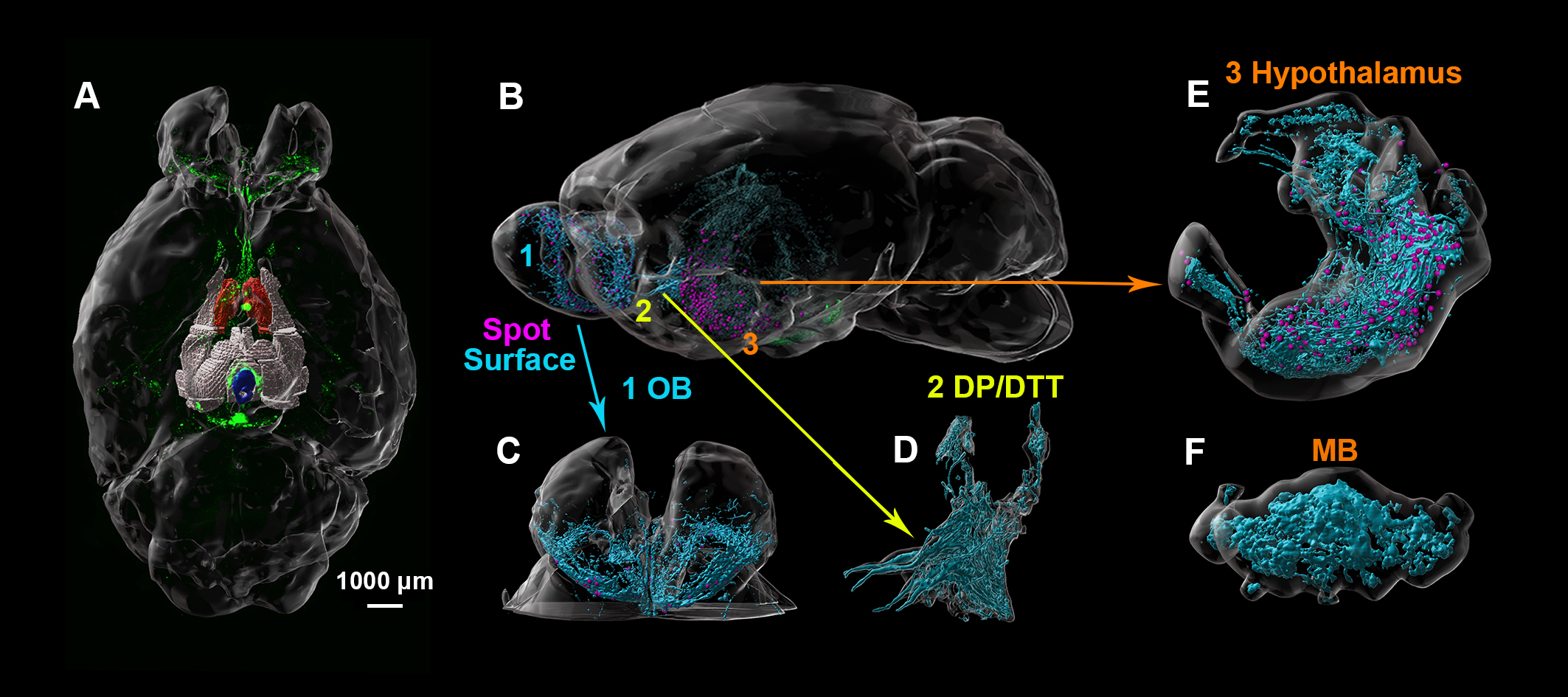


**Fig. S2.** The brain segmentation map obtained by matching the mouse whole brain with the Allen brain atlas (A). The cell body and axon fluorescence signals of GnRH neurons were quantified by the Spots and Surface algorithm of the Imaris software in the whole brain (B), OB (C), DP/DTT (D), and hypothalamus (E) and mammillary body (F).


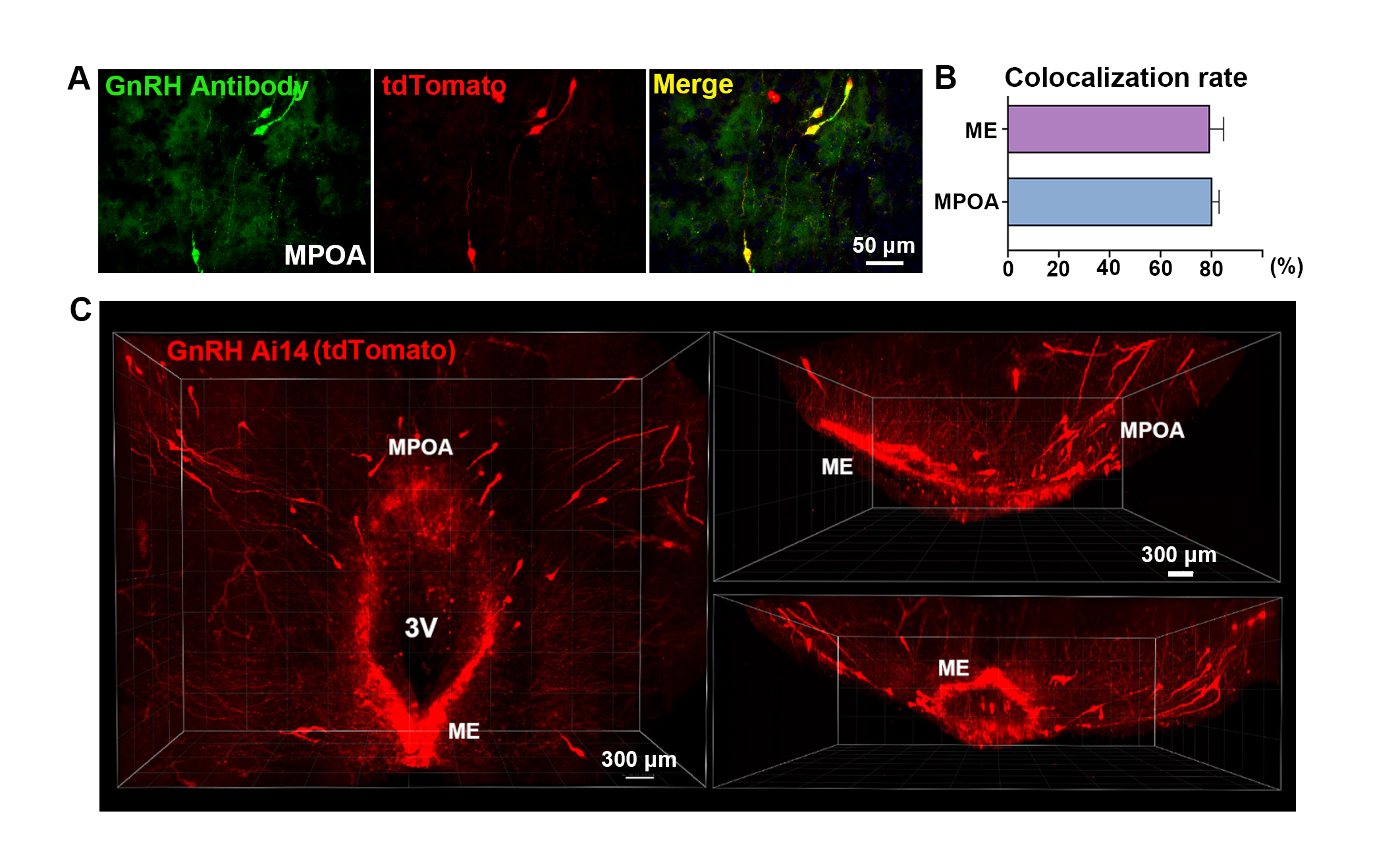


**Fig. S3.** Colocalization of GnRH-Ai14 (tdTomato) with GnRH antibody. (A) Immunofluorescence staining showed GnRH neurons labeled with tdTomato (red) and anti-GnRH antibody (green) in the MPOA region. The merge demonstrated colocalization of the endogenous and antibody-labeled signals. (B) Quantification of the colocalization rate in the ME and MPOA regions, showing high consistency between the two signals. (C) 3D reconstruction of GnRH-Ai14-expressing neurons and their projections in the MPOA and ME regions, with clear visualization of the GnRH axonal pathways.


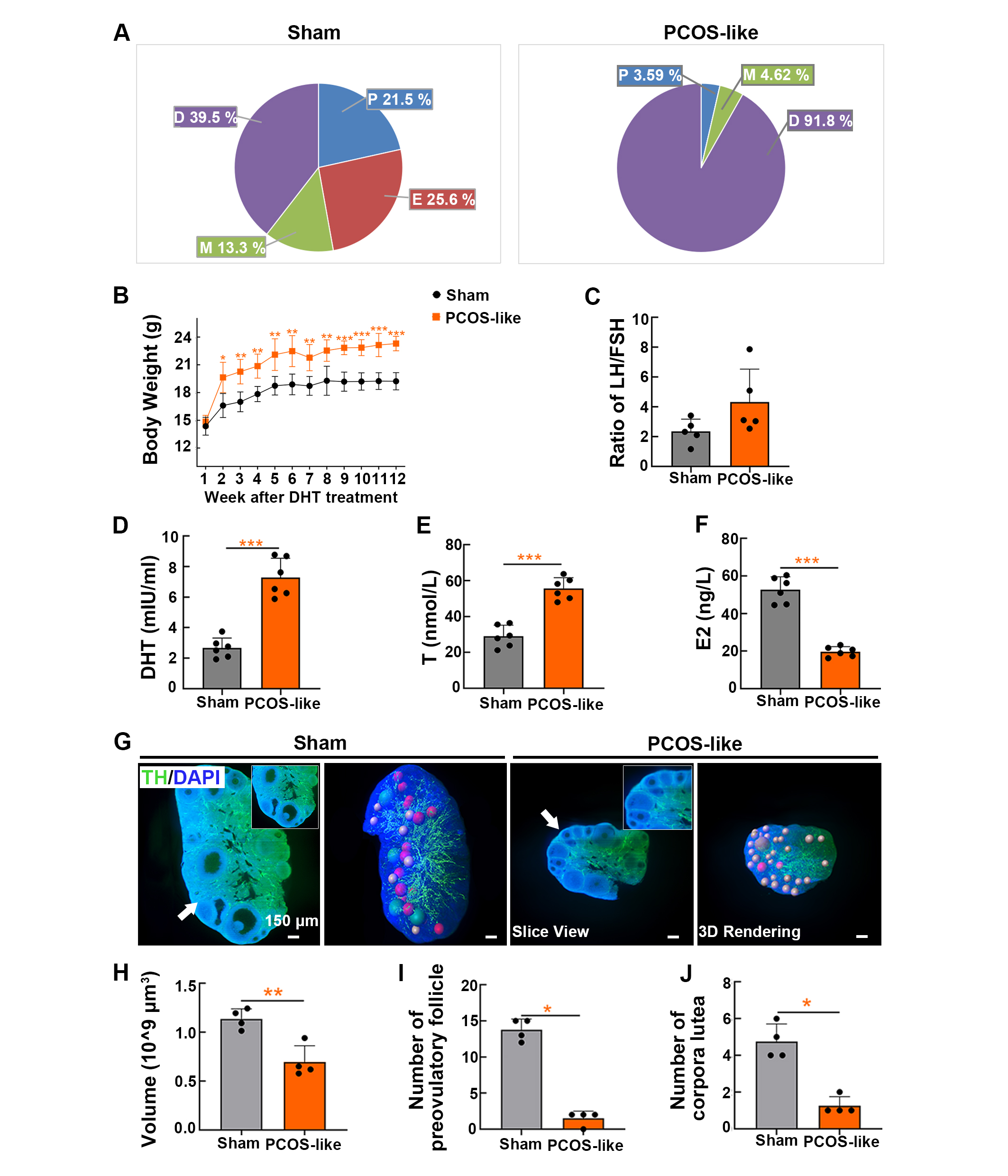


**Fig. S4.** PCOS-like mice exhibited abnormalities in both endocrine and reproductive functions. (A) A quantitative analysis of the cycle stages. Pie charts depicted the percentage of days spent in the different stages of the estrus cycle in sham and PCOS-like mice. (B) The body weights of mice in each group (*n* = 5, unpaired Student’s *t*-test, * *p* <0.05, ** *p* <0.01, *** *p* <0.001). (C-F) Sex hormone expression levels of LH/FSH (C, *n* = 5), DHT (D), T (E), and E2 (F) in the peripheral serum (*n* = 6, unpaired Student’s *t*-test, ****p*<0.001). (G) Reconstructed ovary images stained by antibodies against tyrosine 3-monooxygenase (TH, green) and DAPI (blue) using the iDISCO method. The Spots algorithm of the Imaris software was used to reveal follicle morphology and to count the number of follicles. Corpora lutea (cyan), preovulatory follicles (red), and antral follicles (gray) are indicated. (H-J) Quantitative analysis of ovarian volume, the number of preovulatory follicles, and corpora lutea (*n* = 4, (H) unpaired Student’s *t*-test, ***p*<0.01. (I and J) Mann-Whitney test, **p*<0.05).


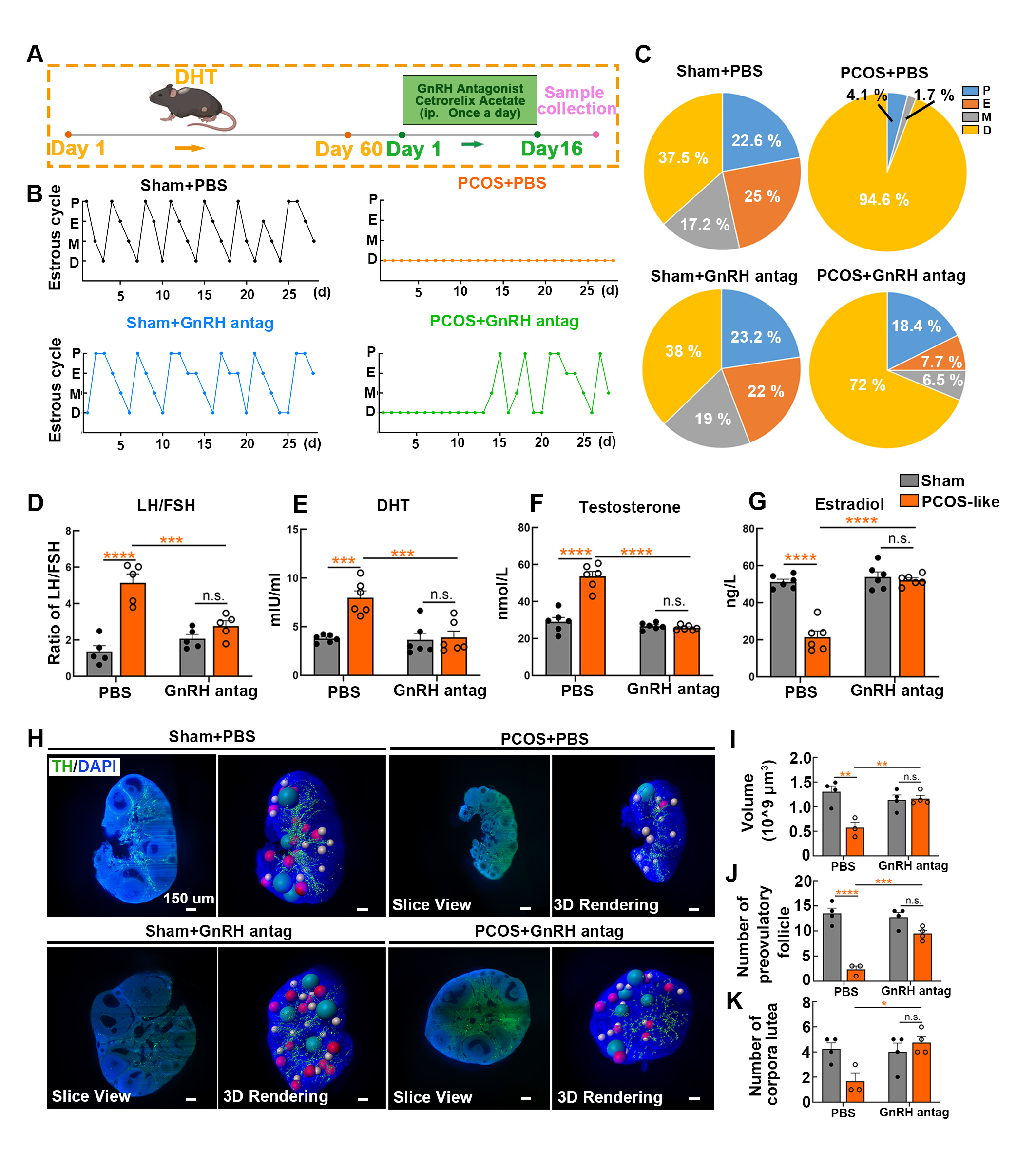


**Fig. S5.** Effects of GnRH antagonist on PCOS-like phenotype induced by DHT. (A) Experimental procedures for DHT and GnRH antagonist administration. Cetrorelix acetate was administered via intraperitoneal injection for 16 days. (B) A representative estrous cycle and (C) pie charts illustrated the distribution of estrous cycle across different groups (*n* = 6, the estrous cycle detection lasted for 28 days). (D-G) Serum levels of LH/FSH (D, *n* = 5), DHT (E), T (F), and E2 (G) in the Sham+PBS, PCOS+PBS, sham+GnRH antagonist, and PCOS+GnRH antagonist group (*n* = 6, two-way ANOVA, Tukey’s post-hoc test. ****p* < 0.001, *****p* < 0.0001). The reconstructed images (H) and quantitative analysis of ovarian volume (I), number of preovulatory follicles (J), and corpora lutea (K) (*n* = 4, two-way ANOVA, Tukey’s post-hoc test. **p* < 0.05, ***p* < 0.01, ****p* < 0.001, and ****p* < 0.001).


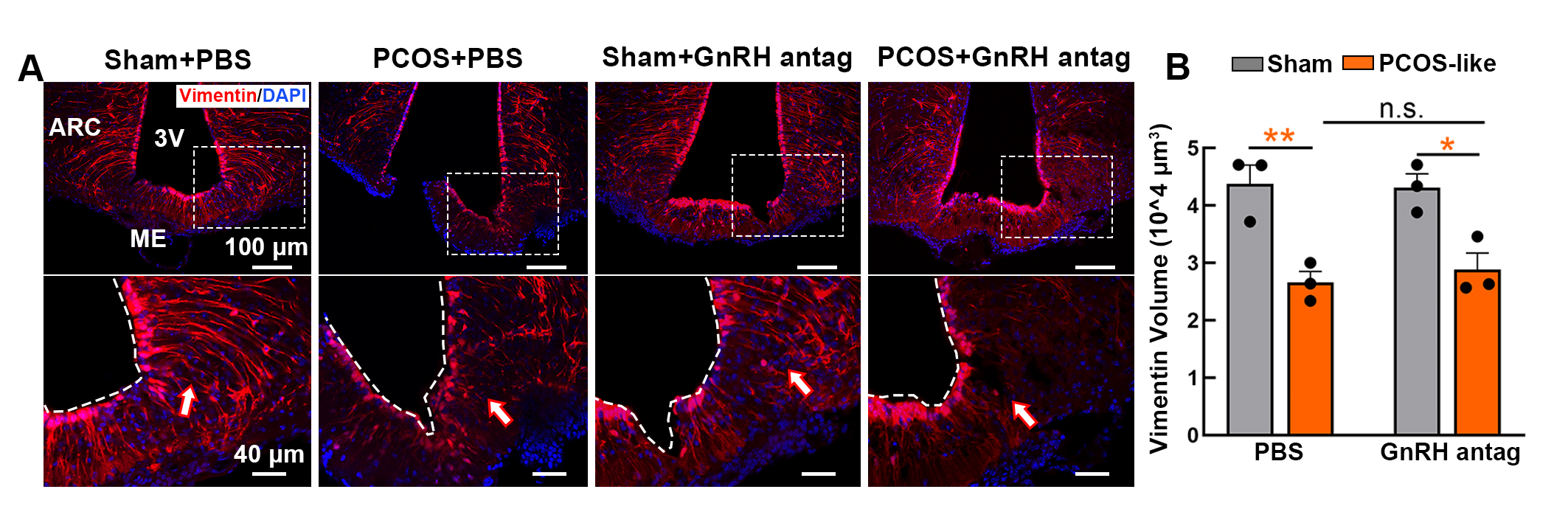


**Fig. S6.** Effects of GnRH antagonist on tanycyte processes in PCOS-like mice. (A) Immunofluorescence images showed vimentin-positive tanycyte processes (red) in the ARC and ME regions in sham and PCOS-like mice treated with PBS or GnRH antagonist. (B) Quantification of vimentin volume in different treatment groups (*n* = 3, two-way ANOVA, Tukey’s post-hoc test.**p* < 0.05, ***p* < 0.01).

**
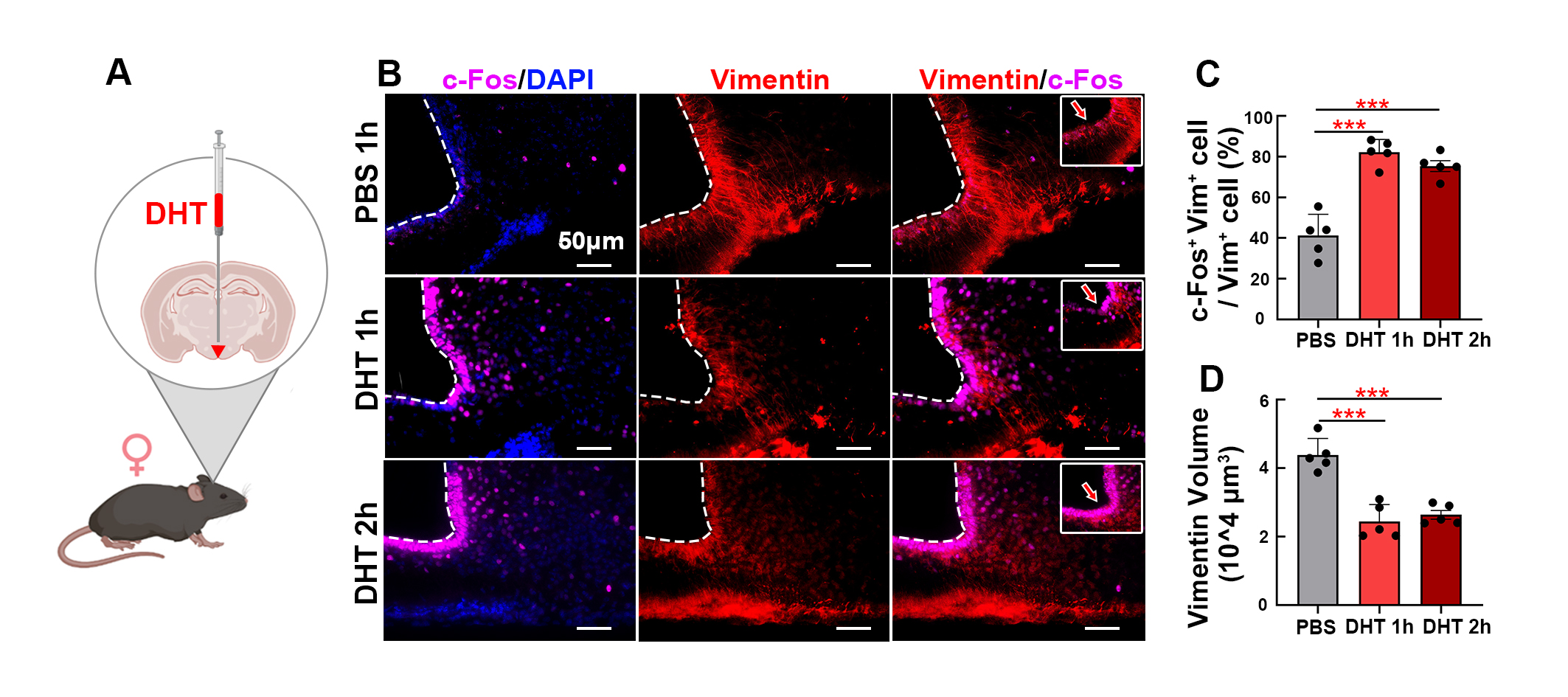
**

**Fig. S7.** Effects of DHT on tanycyte activation and process volume. (A) DHT administration diagram. 1 µL of DHT (10 nM) was injected into the 3V of adult female mice at diestru. (B) Representative immunofluorescence images of c-Fos (magenta) and Vimentin (red) expression in the ARC and ME after DHT or PBS injection. (C) Quantification of the percentage of c-Fos and Vimentin double-positive cells (*n* = 5, one-way ANOVA, Dunnett’s post-hoc test. ****p* < 0.001). (D) Vimentin-positive process volume was significantly reduced in DHT-treated mice compared to PBS controls (*n* = 5, one-way ANOVA, Dunnett’s post-hoc test. ****p* < 0.001).


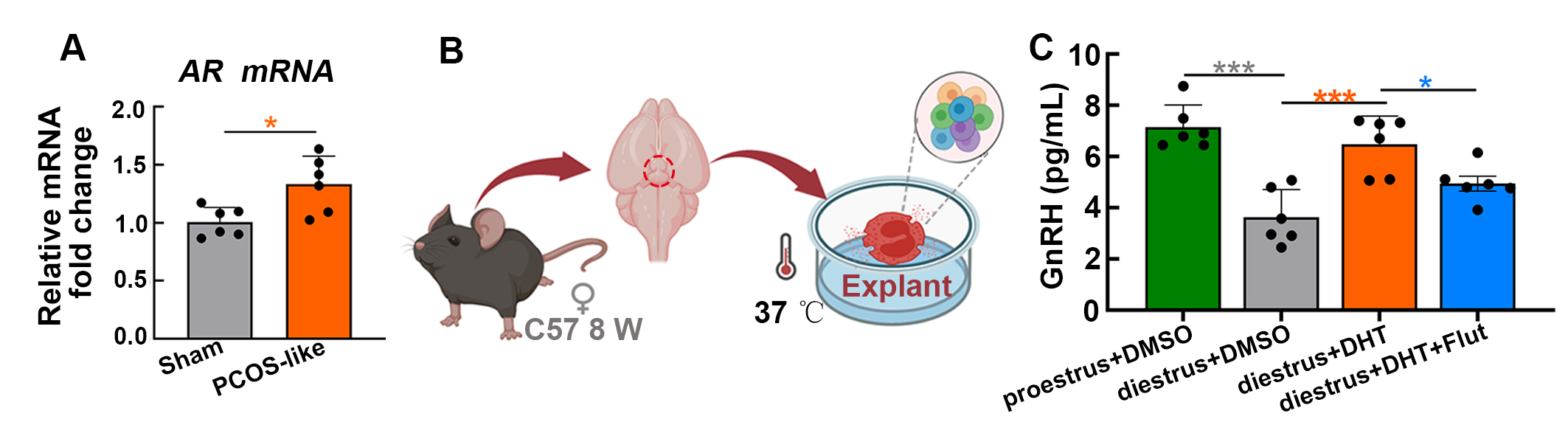


**Fig. S8.** (A) Relative mRNA expression of *AR* in the Sham and PCOS groups (*n* = 6, unpaired Student’s *t-*test, **p*<0.05). (B) Hypothalamic explants were microdissected from adult female mice and cultured in artificial CSF containing DHT and Flut at 37℃. (C) Conditioned medium was collected and processed by GnRH ELISA assay in each group (*n* = 6, one-way ANOVA, Dunnett’s post-hoc test. **p*<0.05, ****p* < 0.001).


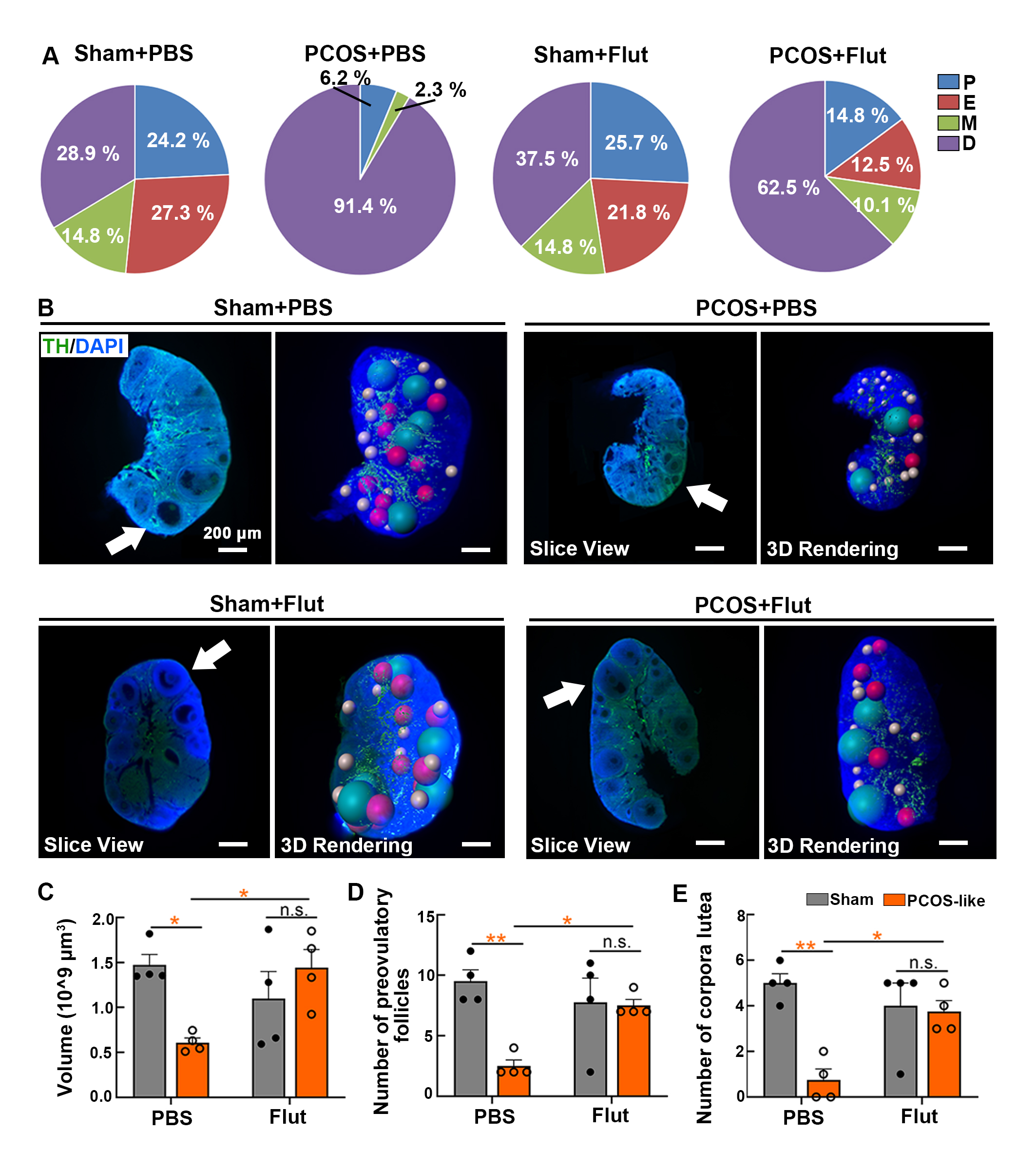


**Fig. S9.** AR antagonists restore ovarian function in PCOS-like mice. (A) Pie charts showed the distribution of estrous cycle stages in different groups. PCOS+PBS mice displayed prolonged diestrus (D), while flutamide treatment (PCOS+Flut) partially restored the estrous cycle with an increase in proestrus (P) and estrus (E) stages. (B) Reconstructed ovary images in the Sham+PBS, PCOS+PBS, Sham+Flut, and PCOS+Flut groups stained by TH (green) and DAPI (blue) antibodies. Corpora lutea (cyan), preovulatory follicles (red), and antral follicles (gray) were identified by the Spots algorithm. (C-E) Quantitative analysis of the ovarian volume, the number of reovulatory follicles, and the number of corpora lutea (*n* = 4, two-way ANOVA, Tukey’s post-hoc test. **p* < 0.05, ***p* < 0.01).


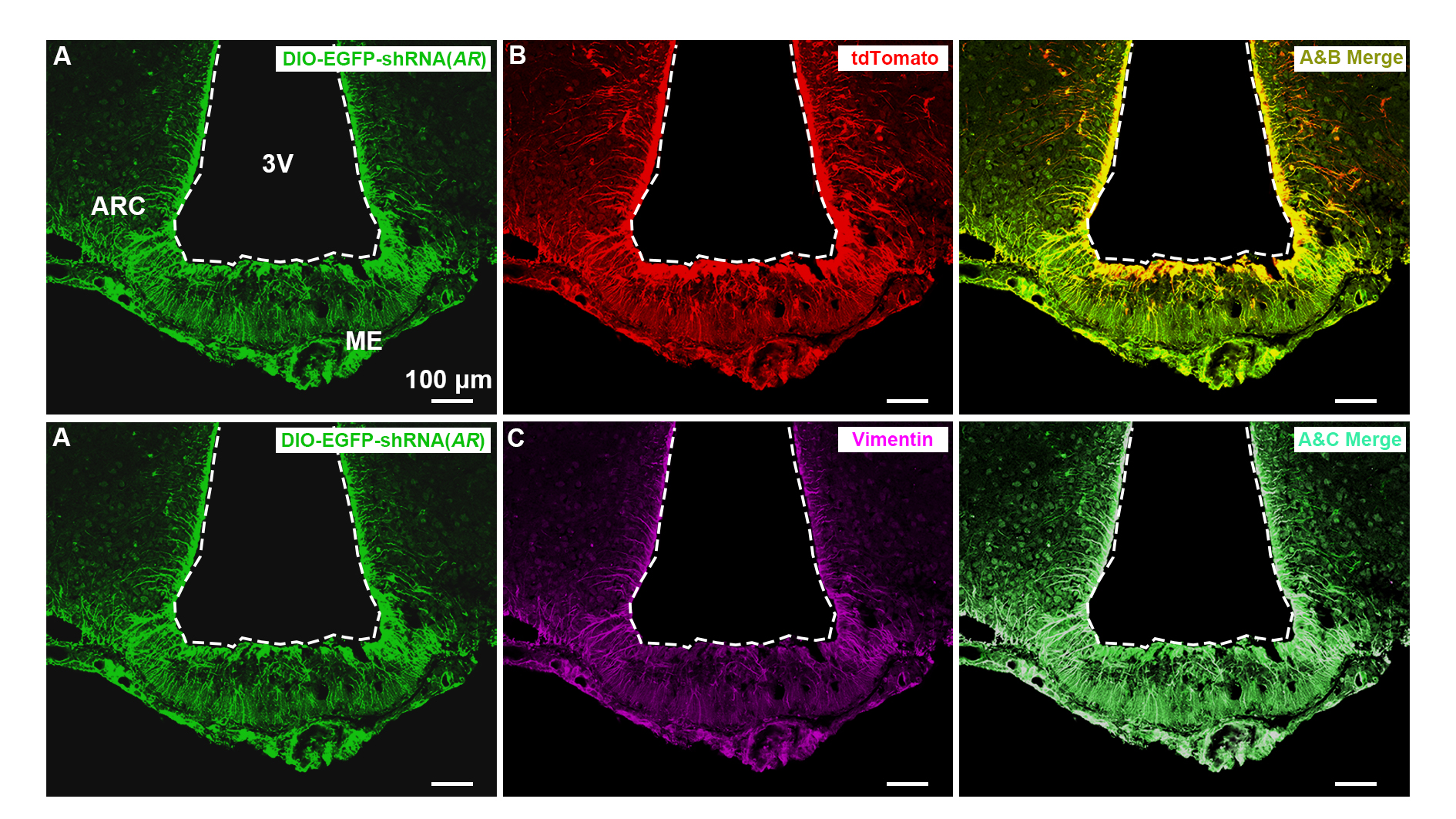


**Fig. S10.** The widespread colocalization of DIO-EGFP-shRNA (*AR*) virus (green), tdTomato (red) carried by Rax-CreERT2;Ai14 mice, as well as the vimentin antibody staining (magenta). The viral infection demonstrated high specificity for tanycytes.

**
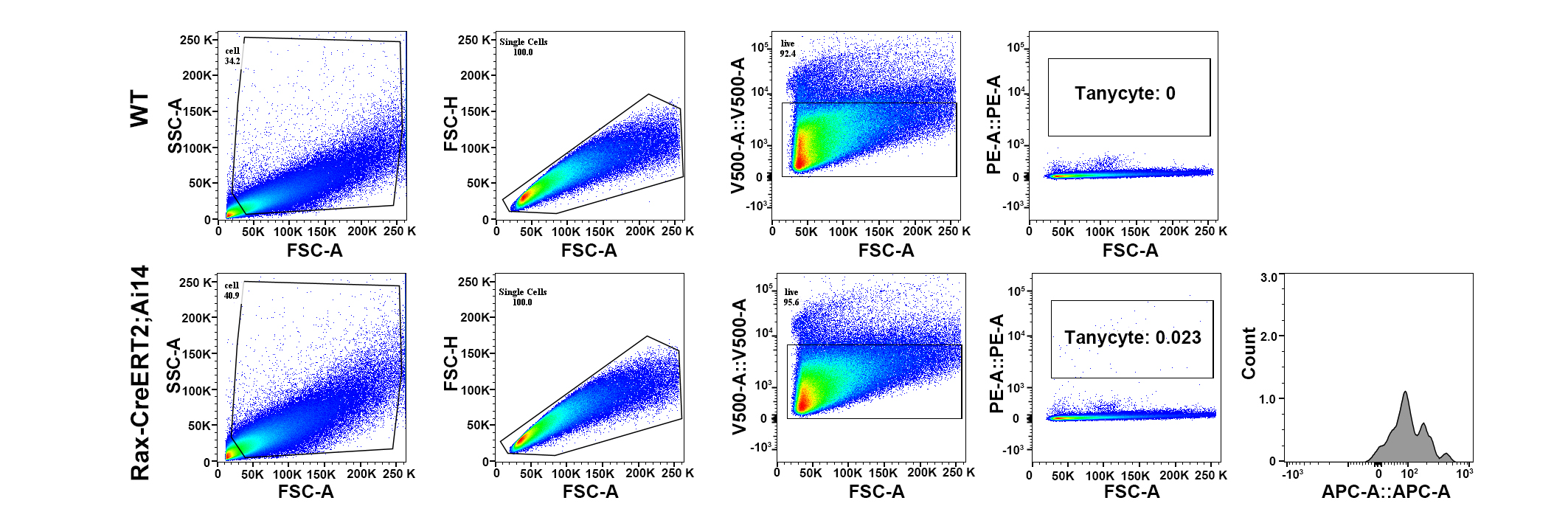
**

**Fig. S11.** Flow cytometry analysis of tanycyte populations in Rax-CreERT2;Ai14 mice. Employing FACS, tdTomato-positive cells within the hypothalamus of Rax-CreERT2;Ai14 mice were isolated to identify tanycytes. The expression levels of AR were quantified via APC pathway. By comparing the proportion of AR-mean fluorescence intensity in tdTomato positive cells, the efficiency of *AR* knockdown by the virus was elucidated.


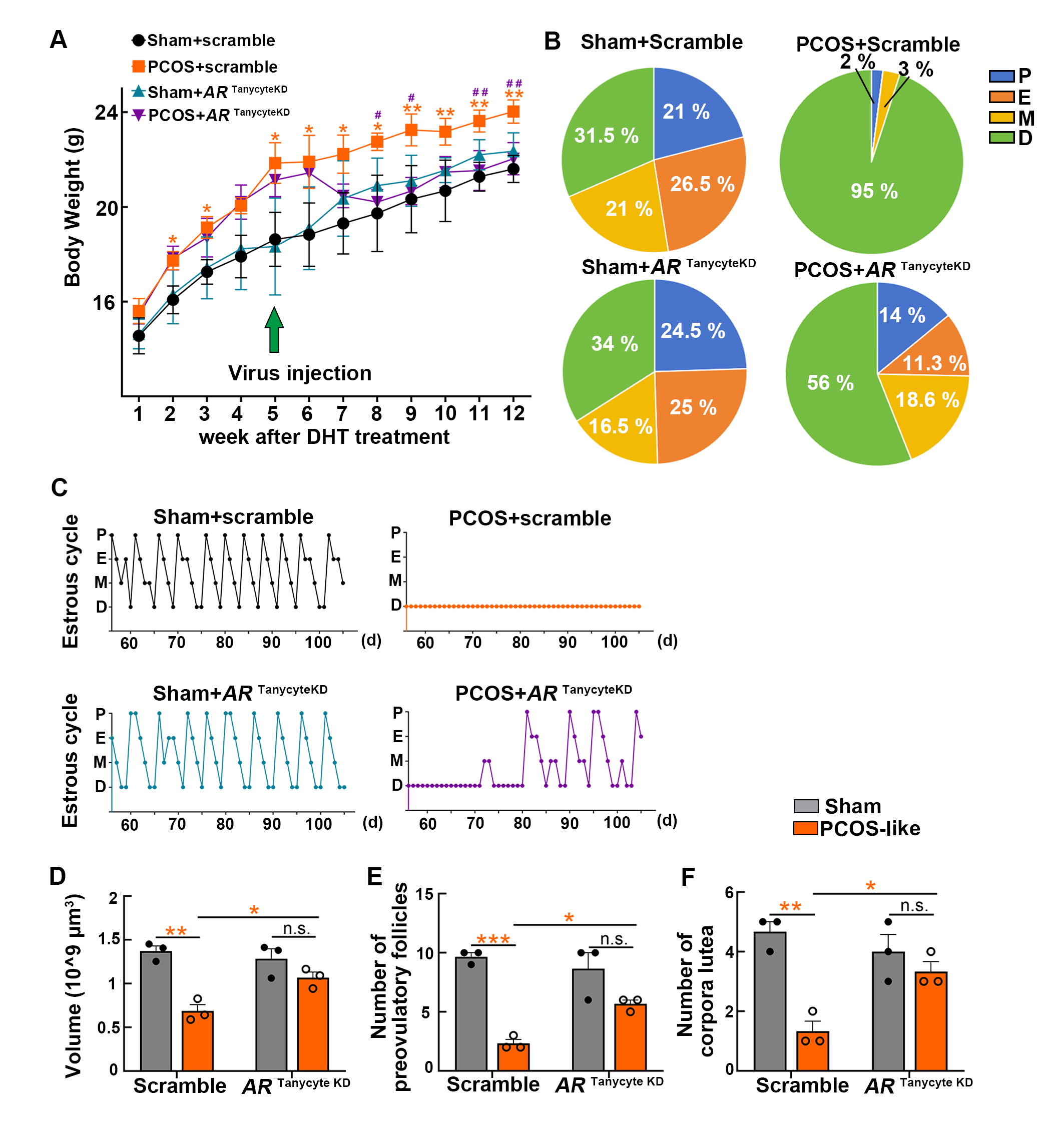


**Fig. S12.** Effects of tanycyte-specific *AR* knockdown on pathological symptoms of PCOS. (A) Body weight changes of mice in each group after the scramble or shAR virus injection (*n* = 3, two-way ANOVA, Tukey’s post-hoc test. **p*<0.05, ***p*<0.01. * *vs.* Sham+scramble group; #*p*<0.05, ##*p*<0.01, # *vs.* PCOS+scramble group). (B and C) Pie charts and representative line chart showed the distribution of estrous cycle stages in different treatment groups. PCOS+Scramble mice exhibited prolonged diestrus, while *AR* knockdown in tanycytes partially restored cyclicity. (D-F) Quantitative analysis of the ovarian volume, the number of preovulatory follicles, and the number of corpora lutea (*n* = 3, two-way ANOVA, Tukey’s post-hoc test. **p* < 0.05, ***p* < 0.01, and ****p* < 0.001).


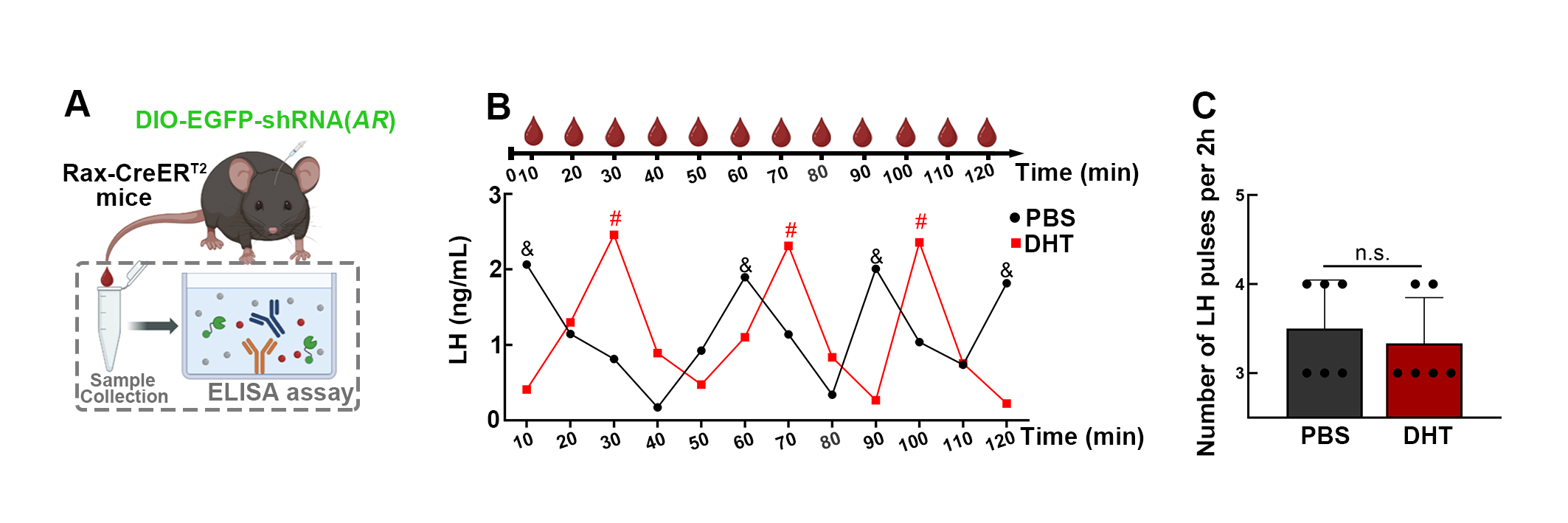


**Figure S13.** (A) Schematic representation of tail-tip blood sample collection in Rax-CreERT2 mice and the ELISA assay. (B and C) Representative graphs and number of LH pulses after receiving PBS and DHT injection (*n* = 6, Mann-Whitney test, & and # indicate the peak value of LH pulses).


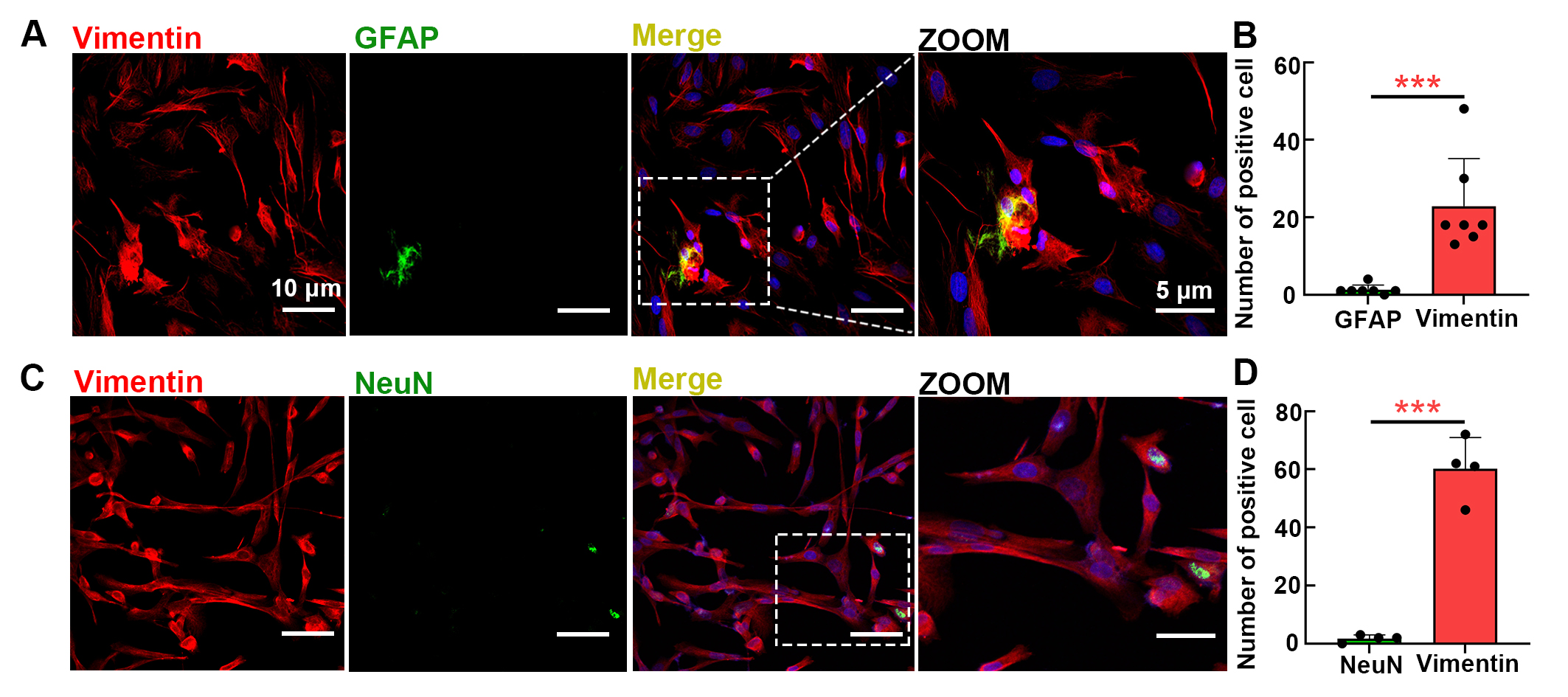


**Fig. S14.** Purity of primary cultured tanycytes. (A) The primary cultured cells were stained with Vimentin (red) and GFAP (green) antibodies, and (B) the number of positive cells was counted (*n* = 7, Mann-Whitney test, ****p* < 0.001). (C) Vimentin-positive cells (red) showed little to no colocalization with the neuronal marker NeuN (green). (D) Quantification demonstrated a significantly higher number of vimentin-positive cells compared to NeuN-positive cells (*n* = 4, unpaired Student’s *t-*test, ****p* < 0.001).


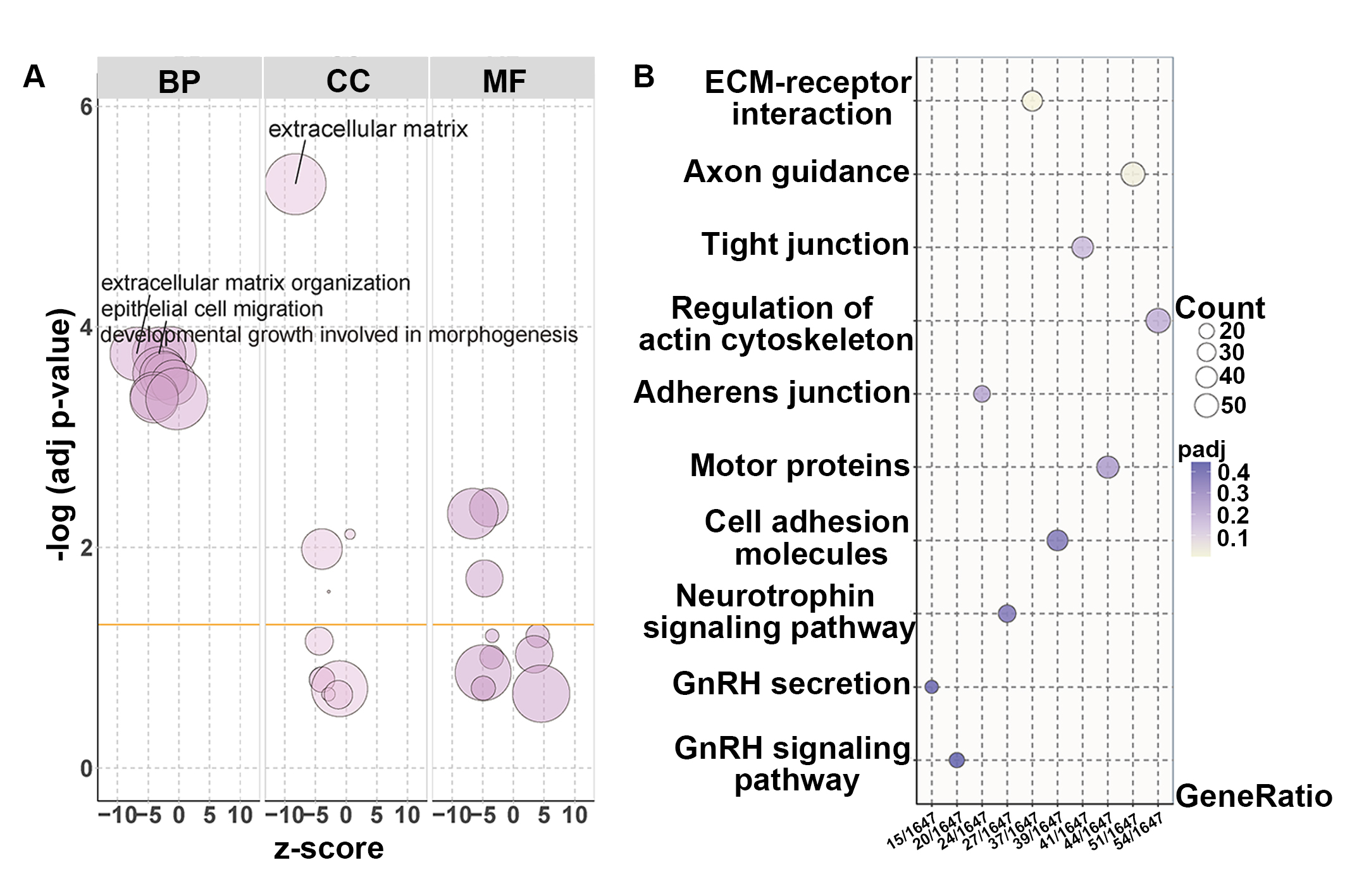


**Fig. S15.** Functional enrichment analysis of differentially expressed genes in tanycytes. (A) Gene Ontology (GO) enrichment analysis showing biological processes (BP), cellular components (CC), and molecular functions (MF) associated with differentially expressed genes. (B) KEGG pathway analysis indicated significant enrichment in pathways related to ECM-receptor interaction, axon guidance, tight junctions, and GnRH signaling.


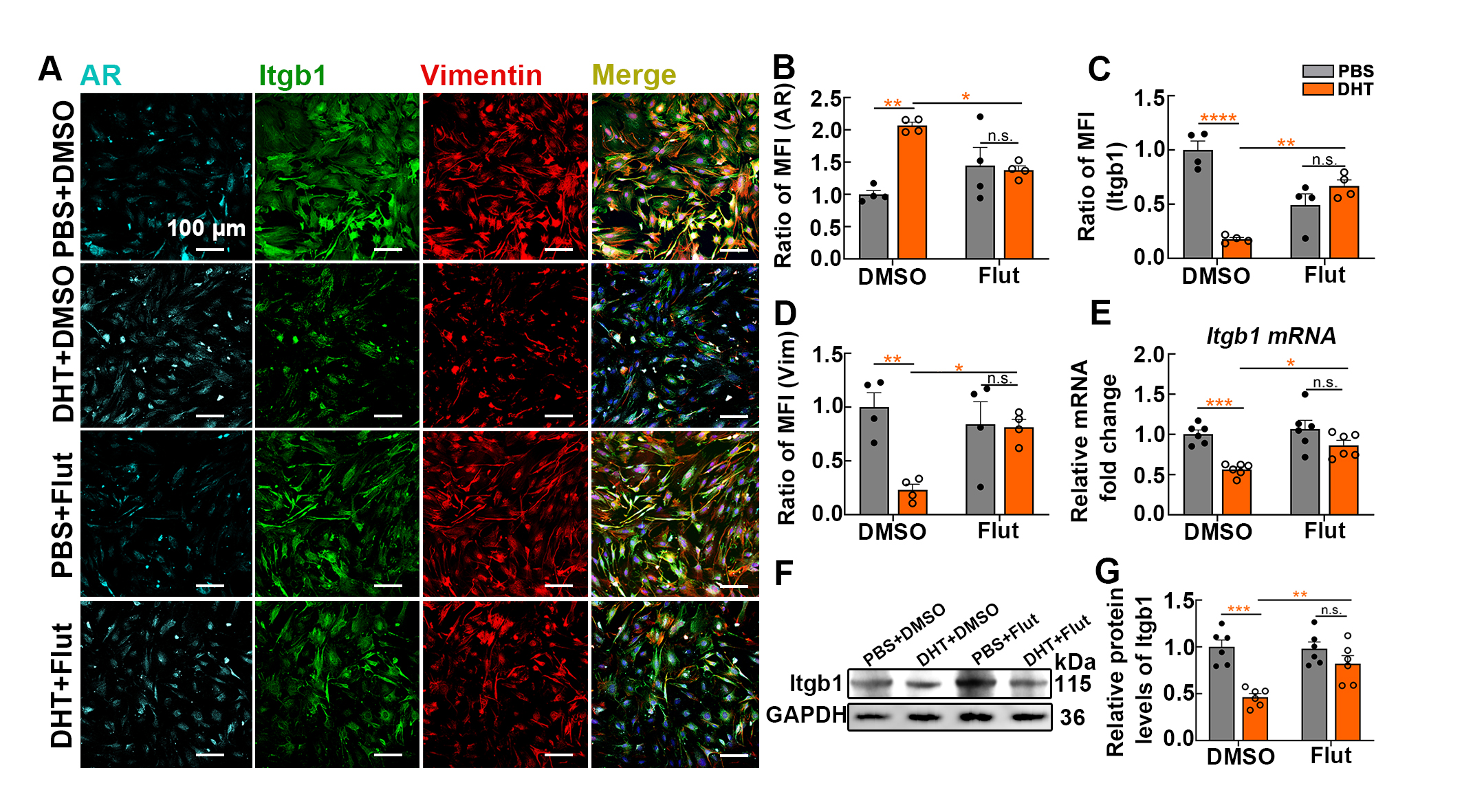


**Fig. S16.** Effects of DHT and flutamide on AR, Itgb1, and Vimentin expression in cultured cells. (A) Representative immunofluorescence images of AR (cyan), Itgb1 (green), and Vimentin (red) expression in primary tanycytes after exposure to DHT and Flut for 48h. (B-D) Quantification of mean fluorescence intensity (MFI) for AR, Itgb1, and Vimentin (*n* = 4, two-way ANOVA, Tukey’s post-hoc test. **p* < 0.05, ***p* < 0.01, and *****p* < 0.0001). (E-G) Real-time PCR analysis and representative western blotting showing the expression of Itgb1 in the primary tanycytes (*n* = 6, two-way ANOVA, Tukey’s post-hoc test. **p* < 0.05, ***p* < 0.01, and ****p* < 0.001).**
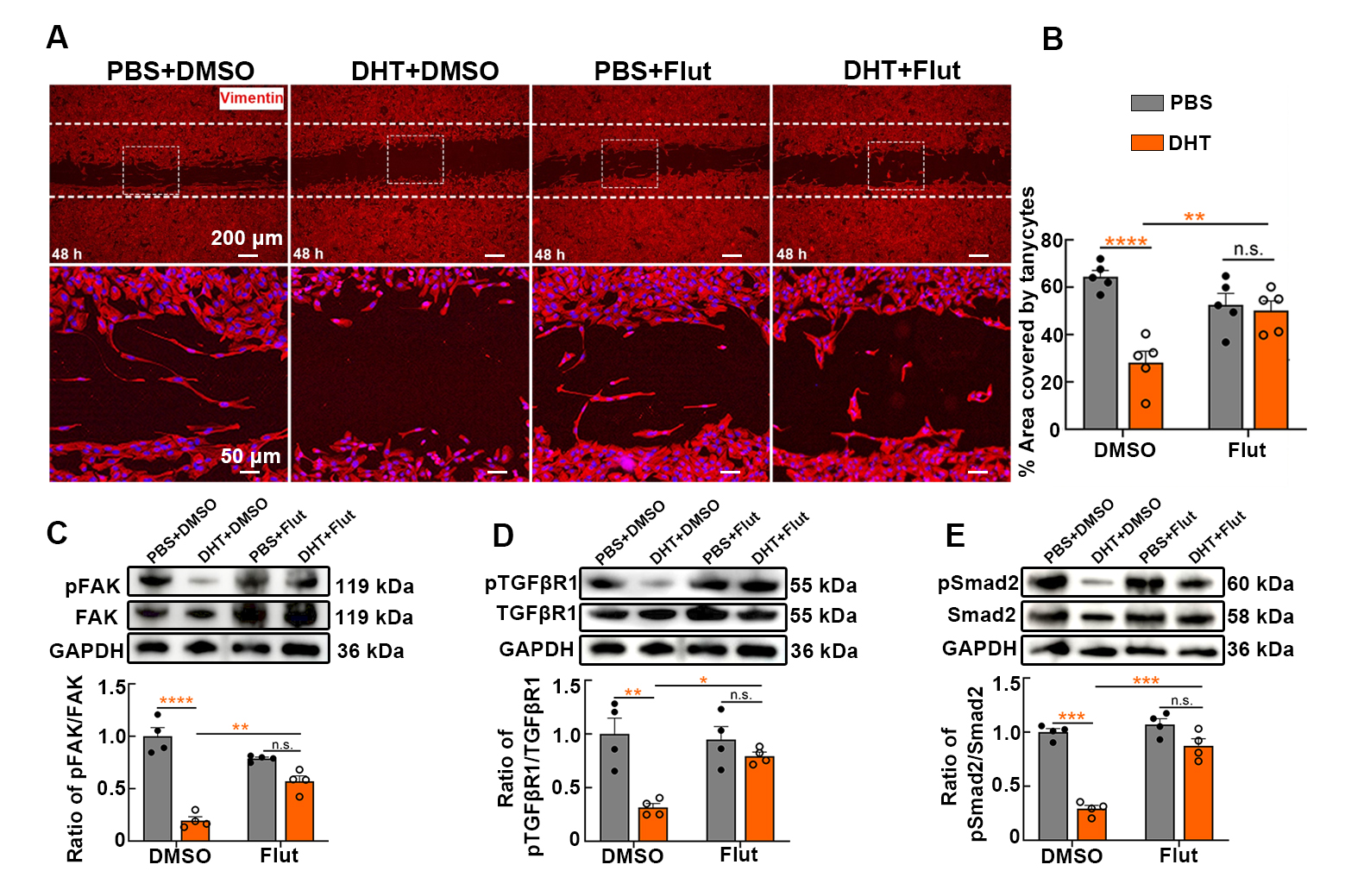
Fig. S17.** (A) Representative images and (B) the area covered by tanycytes in a scratch wound-healing assay performed on primary tanycytes in each group (*n* = 5, two-way ANOVA, Tukey’s post-hoc test. ***p* < 0.01, *****p* < 0.0001). (C-E) The representative immunoblots bands and quantitative analysis of FAK, TGF-βR1, and Smad2 (*n* = 4, two-way ANOVA, Tukey’s post-hoc test. **p* < 0.05, ***p* < 0.01, ****p* < 0.001, and *****p* < 0.0001).

**
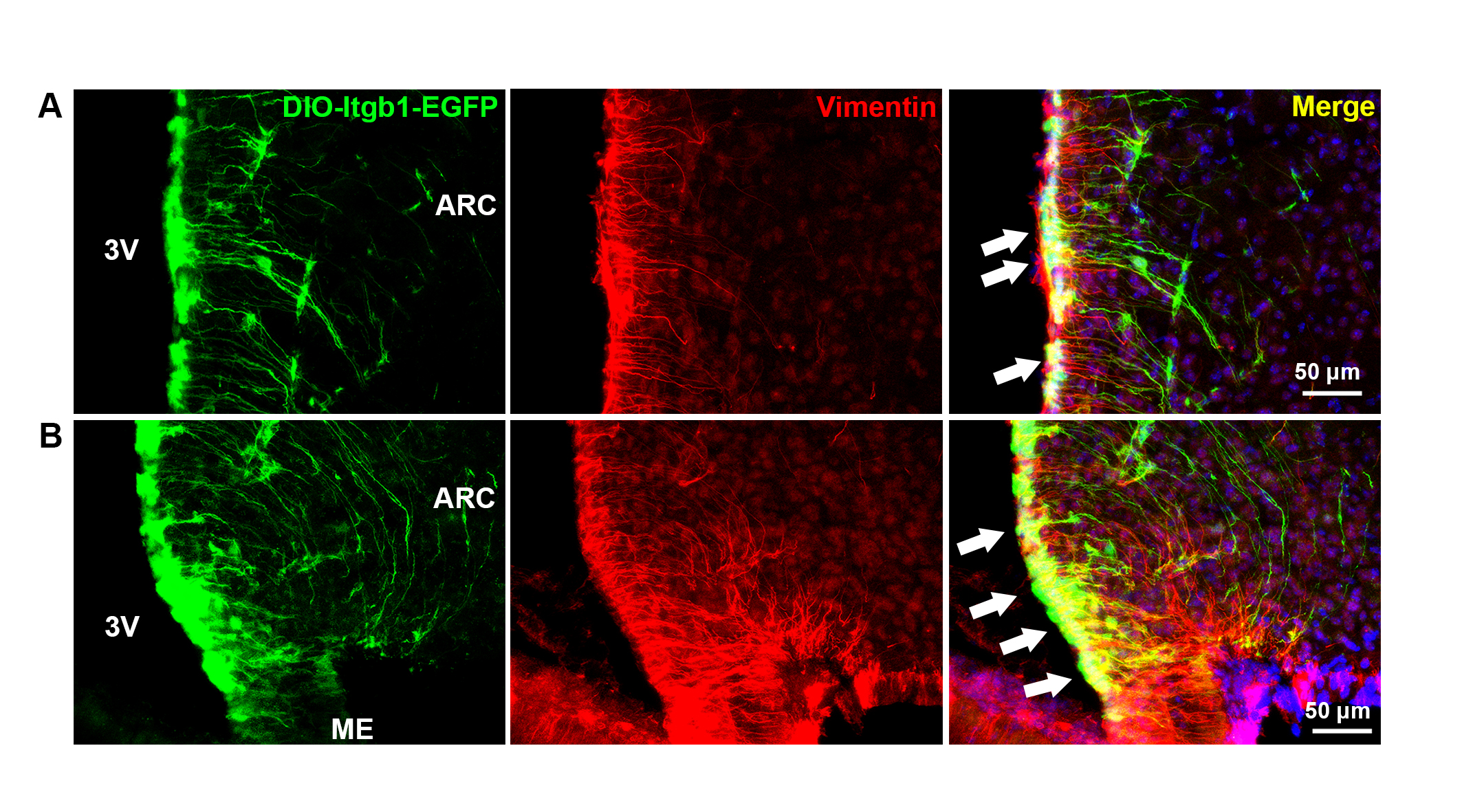
**

**Fig. S18.** Co labeling of Itgb1 specific overexpression virus (green) and vimentin antibody staining (red) near hypothalamic ARC and ME. The virus has effectively infected the tanycyte cell body and processes.


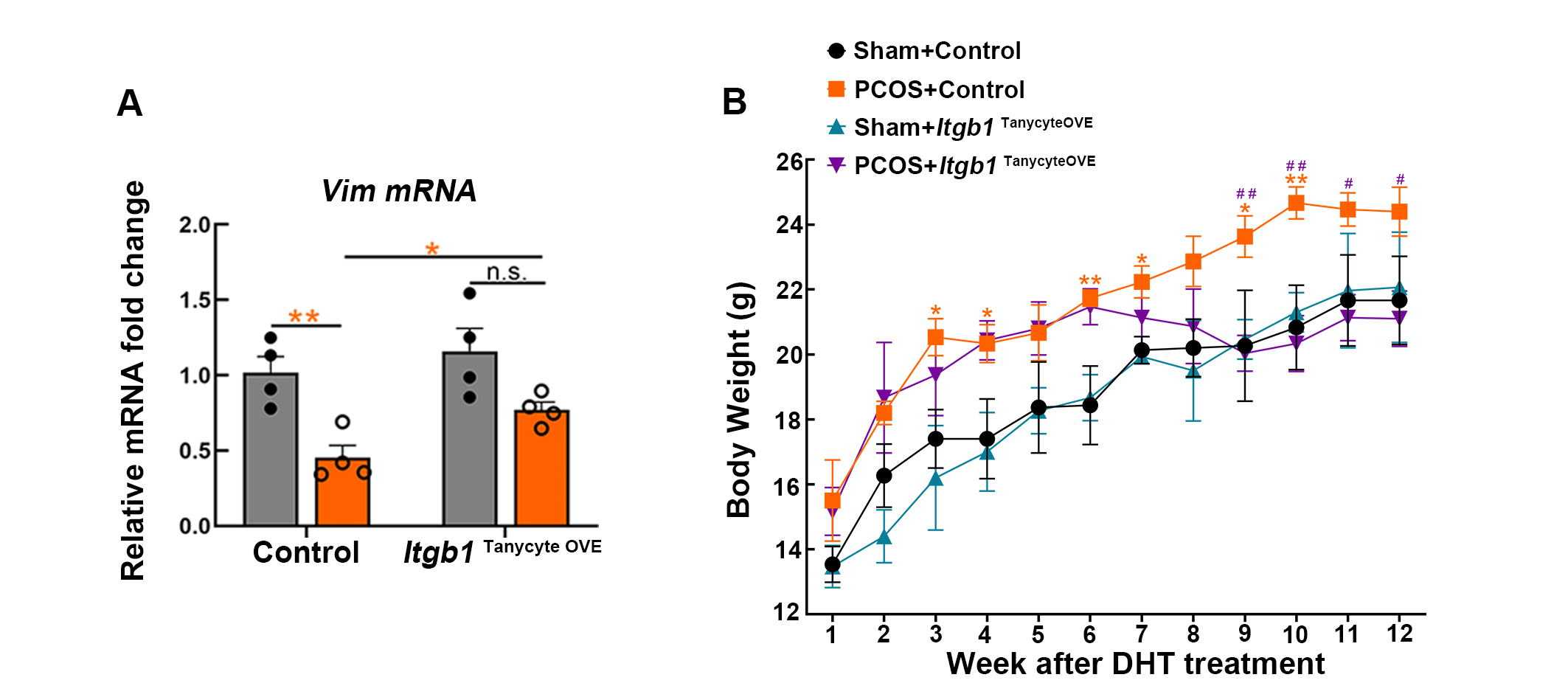


**Fig. S19.** (A) Expression levels of *Vimentin* mRNA (*n* = 4, two-way ANOVA, Tukey’s post-hoc test. **p* < 0.05, ***p* < 0.01). (B) Weight changes of mice in each group after DHT and virus injection (*n* = 3, two-way ANOVA, Tukey’s post-hoc test. **p*<0.05, ***p*<0.01. * *vs.* Sham+scramble group; #*p*<0.05, # #*p*<0.01, # *vs.* PCOS+scramble group).

**
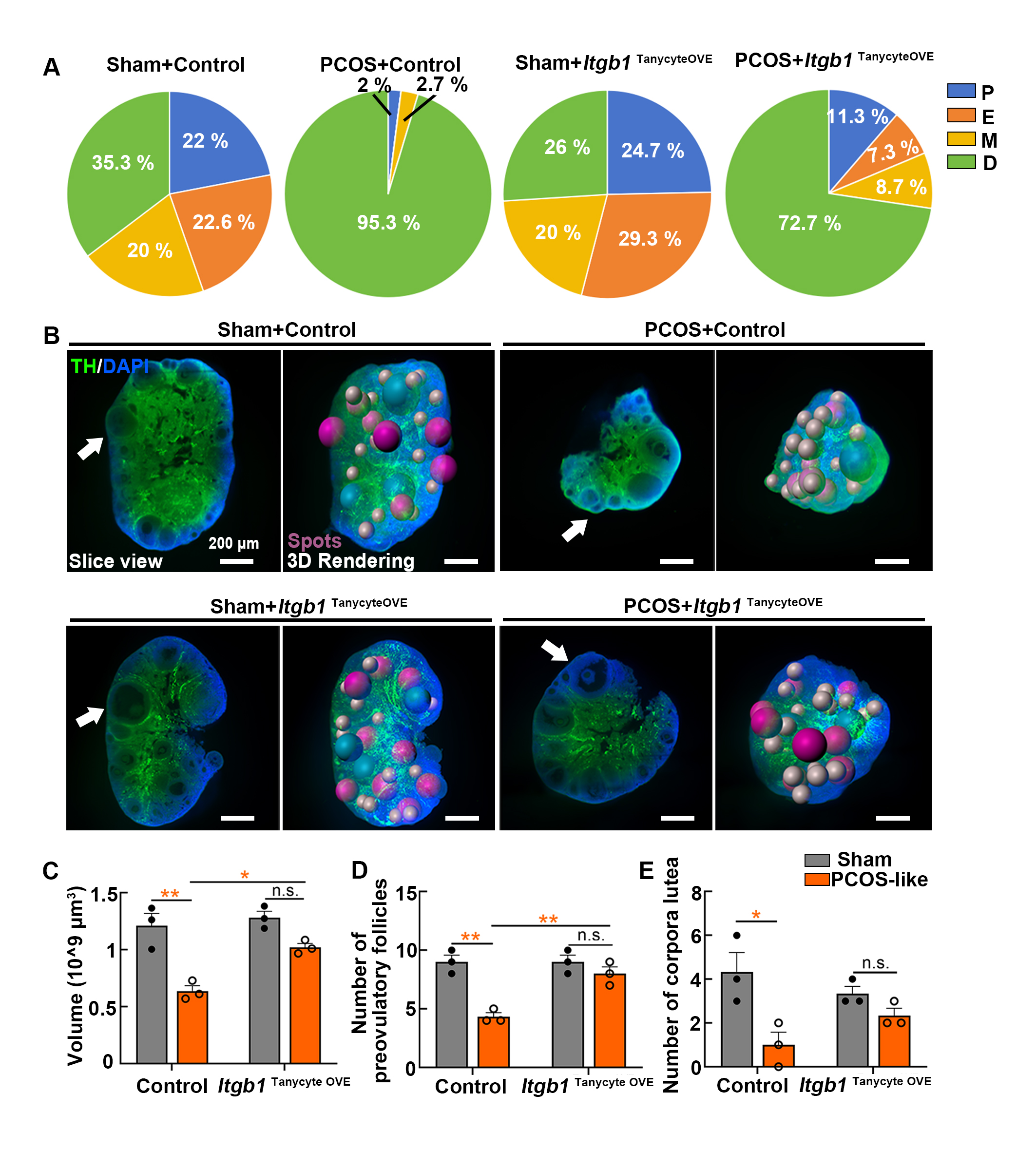
Fig. S20.** Effects of *Itgb1* overexpression in tanycytes on estrous cycle and ovulation function in PCOS-like mice. (A) Pie charts represented the distribution of estrous cycle stages in different treatment groups. PCOS+Control mice exhibited prolonged diestrus, while *Itgb1* overexpression (*Itgb1* ^Tanycyte OVE^) partially restored cyclicity. (B) Three-dimensional images and reconstructed follicles of mouse ovaries in each group. Corpora lutea (cyan), preovulatory follicles (red), and antral follicles (gray) were identified by the Spots algorithm. (C-E) The ovarian volume (C), the number of pre-ovulatory follicles (D) and corpus luteum (E) were quantified using iDISCO method and analyzed with Imaris software (*n* = 3, two-way ANOVA, Tukey’s post-hoc test. **p* < 0.05, ***p* < 0.01).
